# Impacts of the iCanCope Digital Pain Self-Management Program Among Adolescents and Young Adults With Chronic Pain: A Randomized Controlled Trial

**DOI:** 10.1080/24740527.2026.2717605

**Published:** 2026-10-15

**Authors:** Chitra Lalloo, Fareha Nishat, Tonya Palermo, Lori Montgomery, Fiona Campbell, Krista Baerg, Jill Chorney, Bruce Dick, Tania Di Renna, Karim Mukhida, Melanie Noel, Patricia Poulin, Mark Simmonds, Ramesh Zacharias, Lauren Harris, Cleo Davies-Chalmers, Carley Ouellette, Vina Mohabir, Quynh Pham, Joe Cafazzo, Sarah Brennenstuhl, Brenna McDermott, Rachel Kelly, Jennifer N. Stinson

**Affiliations:** a The Hospital for Sick Children, Child Health Evaluative Sciences, Toronto, ON, Canada; b Institute of Health Policy, Management & Evaluation, University of Toronto, Toronto, ON, Canada; c Center for Child Health, Behavior and Development, Seattle Children’s Research Institute, Seattle, Washington, USA; d Department of Anesthesiology and Pain Medicine, University of Washington School of Medicine, Seattle, Washington, USA; e Departments of Family Medicine and Anesthesiology, Peri-operative and Pain Medicine, University of Calgary, Calgary, Alberta, Canada; f Department of Anesthesia and Pain Medicine, Hospital for Sick Children, Toronto, Ontario, Canada; g Department of Pediatrics, University of Saskatchewan, Saskatoon, Saskatchewan, Canada; h Centre for Pediatric Pain Research, IWK Health, Halifax, Nova Scotia, Canada; i Department of Anesthesiology and Pain Medicine, University of Alberta, Edmonton, Alberta, Canada; j Toronto Academic Pain Medicine Institute, Women’s College Hospital, Toronto, Ontario, Canada; k Department of Anesthesia, Pain Management & Perioperative Medicine, Dalhousie University, Halifax, Nova Scotia, Canada; l Alberta Children’s Hospital, Hotchkiss Brain Institute, University of Calgary, Calgary, AB, Canada; m Department of Anesthesiology & Pain Medicine, University of Ottawa, Ottawa, ON, Canada; n Department of Psychology, The Ottawa Hospital, Ottawa, ON, Canada; o Clinical Epidemiology Program, The Ottawa Hospital Research Institute, Ottawa, ON, Canada; p Michael G. DeGroote Pain Clinic, Hamilton Health Sciences, Hamilton, Ontario, Canada; q School of Nursing, McMaster University, Hamilton, Ontario, Canada; r Center for Digital Therapeutics, Toronto General Hospital, University Health Network, Toronto, Ontario, Canada; s Lawrence S. Bloomberg Faculty of Nursing, University of Toronto, Toronto, Ontario, Canada

**Keywords:** Chronic pain, self-management, smartphone, adolescents, mHealth, digital therapeutics

## Abstract

iCanCope™ (ICC) is a digital self-management program for 15–25 year olds with chronic pain. This study evaluated program satisfaction and efficacy through a two arm randomized controlled trial in Canadian tertiary care pain clinics. Participants were randomized to receive either ICC (app and website with symptom tracking and trending, pain education, goal setting, library, community forum) or an identically branded active control program with limited features. Outcomes were assessed at baseline, postprogram (2 months; T2), and follow-up (6 months; T3). Satisfaction was assessed using the Acceptability eScale and qualitatively with a subset of intervention participants. Linear mixed effects models characterized treatment effects on primary (pain interference) and secondary outcomes. Of 302 enrolled participants, 297 were analyzed (mean age = 17.1 ± 2.4, 83% female). Outcome completion rates were 89% at T2 and 71% at T3. Program acceptability was high in both the intervention (33.9 ± 7.0) and control group (32.7 ± 6.8). Most participants exhibited moderate to high engagement with daily checkins (intervention: 79%, active control: 80%). No significant treatment effects were found. However, improvements across time in pain interference at T2 (β = −0.58, *p* < .001) and T3 (β = −0.64, *p* = .0016) and improvements in knowledge about managing pain at T2 (β = −0.54, *p* < .0001) and T3 (β = −1.04, *p* < .0001) were found for both groups. Most (15/16) interviewees reported that ICC enhanced their confidence and/or ability to engage in pain self-management. Digitally mediated symptom tracking and trending and pain education is engaging and beneficial for 15–25 year olds with chronic pain. Use of an active-control may have diminished the ability to detect treatment-group effects. ICC has been translated to French-Canadian and publicly released as a free self-management tool. **Trial Registration**: ClinicalTrials.gov, NCT02601755

## RÉSUMÉ

iCanCope™ (ICC) est un programme numérique d’autoprise en charge destiné aux personnes âgées de 15 à 25 ans vivant avec une douleur chronique. Cette étude a évalué la satisfaction à l’égard du programme et son efficacité dans le cadre d’un essai contrôlé randomisé à deux bras, mené dans des cliniques de la douleur offrant des soins tertiaires au Canada. Les participants ont été répartis aléatoirement pour recevoir soit le programme ICC (application et site Web permettant de suivre les symptômes et leur évolution, de s’informer sur la douleur et de se fixer des objectifs, et donnant accès à une bibliothèque et à un forum communautaire), soit un programme témoin actif présenté de la même manière, mais doté de fonctionnalités limitées. Les critères de jugement ont été évalués au début de l’étude, à la fin du programme (2 mois; T2) et lors du suivi (6 mois; T3). La satisfaction a été évaluée à l’aide de l’Acceptability eScale ainsi que par une démarche qualitative auprès d’un sous-groupe de participants ayant reçu l’intervention. Des modèles linéaires à effets mixtes ont permis de caractériser les effets du traitement sur le critère de jugement principal (interférence de la douleur) et les critères secondaires. Sur les 302 participants inscrits à l’étude, 297 ont été inclus dans l’analyse (âge moyen : 17,1 ± 2,4 ans, 83 % de femmes). Les taux de réponse aux évaluations étaient de 89 % à T2 et de 71 % à T3. L’acceptabilité du programme était élevée tant dans le groupe d’intervention (33,9 ± 7,0) que dans le groupe témoin (32,7 ± 6,8). La plupart des participants ont présenté un degré d’engagement modéré à élevé dans le suivi quotidien (groupe d’intervention : 79 %; groupe témoin actif : 80 %). Aucun effet significatif du traitement n’a été observé. Toutefois, dans les deux groupes, l’interférence de la douleur s’est atténuée au fil du temps, comme en témoignent les résultats à T2 (β = -0,58, p < 0,001) et à T3 (β = -0,64, p = 0,0016), et les connaissances sur la prise en charge de la douleur se sont améliorées à T2 (β = −0,54, p < 0,0001) et à T3 (β =−1,04, p < 0,0001). La plupart des personnes interrogées (15/16) ont déclaré que l’ICC avait renforcé leur confiance, leur capacité à prendre elles-mêmes en charge leur douleur, ou les deux. Le suivi numérique des symptômes et de leur évolution ainsi que l’éducation sur la douleur suscitent l’intérêt des personnes de 15 à 25 ans vivant avec une douleur chronique et leur sont bénéfiques. Le recours à un groupe témoin actif pourrait avoir limité la capacité à détecter les effets du traitement. L’ICC a été traduit en français canadien et mis gratuitement à la disposition du public comme outil d’autoprise en charge.

## Background

Chronic pain in adolescents and young adults (AYA) is increasingly common, with prevalence estimates of 20% or >1 million Canadian AYA reporting pain lasting longer than 3 months.^1,2^ Unrelieved or undertreated chronic pain can negatively affect all aspects of health-related quality of life (HRQL) and interfere with normative developmental tasks. These impacts span multiple domains, including physical (e.g., activity limitations, poor sleep, fatigue), emotional (e.g., anxiety, depression, stress), social (e.g., restrictions in peer, intimate, and family relationships), cognitive (e.g., academic difficulties, impaired attention and memory), and role functioning (e.g., school and work absenteeism).^3^ Moreover, the AYA period represents a critical developmental stage, offering a unique opportunity to establish positive health behaviors, prepare for transition to adult healthcare, and potentially minimize future pain-related disability.^4,5^

Chronic pain management is guided by the biopsychosocial model, which emphasizes the integration of pharmacological, physical, and psychological strategies (i.e., “the 3 P’s”).^6,7^ Effective implementation of a pain care plan also relies heavily on self-management, defined as *“an individual’s ability to manage the symptoms, treatment, physical and psychological consequences, and lifestyle changes inherent to living with a chronic illness.”*^8,9^ AYA are expected to assume increasing responsibility for their disease management as they grow into young adulthood.^5,9^ However, building and practicing these skills can be challenging amidst other major life changes such as increasing academic demands, shifts in social relationships, the pursuit of independence, and transition from pediatric to adult healthcare.^5,9^ Moreover, traditional pain self-management programs often require frequent in-person sessions, rigid scheduling, and sustained engagement, which may not align with the health needs and routines of AYA.^10^ These barriers underscore the need for accessible, flexible, and developmentally appropriate co-designed self-management programs tailored to the realities of AYA living with chronic pain.

Existing evidence suggests that cognitive behavioral therapy (CBT) is among the most effective approaches for supporting self-management in AYA with chronic pain, improving coping skills, enhancing HRQL, and reducing healthcare use across a range of conditions.^11,12^ Embedding CBT principles into accessible and engaging formats may help overcome barriers to participation and promote early integration of self-management skills into the care trajectory of AYA.

Digital technologies offer a compelling mechanism for delivering education and psychosocial support that is tailored for AYA with chronic pain. Smartphones are deeply integrated into daily routines, offering a platform for scalable, AYA-friendly interventions. However, while many pain-related apps exist, few are evidence-based and, to our knowledge, none are specifically designed with and for AYA.^13^ This age group warrants focused attention, as AYA have been underrepresented in intervention-based research, despite contributing to a growing proportion of individuals living with chronic pain.^14^ Moreover, AYA are particularly vulnerable to disruptions in care during the transition from pediatric to adult care, increasing their risk of unmet pain management needs.^5^

As previously described, our group developed a digital pain self-management platform called *iCanCope with Pain* (iCanCope; https://www.icancope.ca/) through a phased, user-centered approach.^15,16^ First, qualitative needs assessments were conducted with AYA living with chronic pain and their healthcare providers. These studies identified the pain self-management needs of this group and generated a core program architecture to begin addressing these needs.^16^ Second, iterative co-design methods were used to generate low- and higher‑fidelity prototypes to demonstrate intended program function.^16^ Third, cycles of usability testing were completed to ensure the program was intuitive to use by the target audience of 15–25 year olds with chronic pain.^16^ Patient partners (i.e., AYA with lived experience of chronic pain) were involved in all stages as members of the project team, from ideation to study design to data collection, analysis, interpretation, and knowledge translation.

The iCanCope program is delivered through a smartphone app (iOS/Android) and a complementary education website. The core program feature is symptom tracking and trending, implemented through daily check-ins for pain intensity, mood, sleep, physical activity, and energy levels. Additional features include a library of CBT-based skills training, personalized goal setting, and a community forum.

In the present study, we aimed to evaluate the impacts of the iCanCope program on pain intensity and interference, self-efficacy, emotional wellbeing, sleep function, social functioning, health-related quality of life (HRQL), and user satisfaction. Specifically, iCanCope was compared to an identically branded active-control program that included symptom tracking and trending and pain education but lacked the additional library, goal-setting, and community forum features. This comparison was chosen to assess the incremental value of these added components within the context of digital pain self-management behaviors of AYA, with the hypothesis that the iCanCope intervention would lead to greater reductions in pain-related outcomes and greater improvement in additional outcomes (self-efficacy, emotional wellbeing, sleep, social function, and HRQL) compared to the active-control. The use of an active comparator was also intended to align with design guidelines for digital health trials and was informed by patient-partner discussions highlighting the importance of all participants deriving potential benefit from taking part in the research study.^17–19^

## Methods

### Trial design

A two-arm RCT with a 1:1 allocation ratio with repeated measures at baseline, immediately post-intervention, and long-term follow-up was used to assess the impacts of the iCanCope program in AYA with chronic pain. The iCanCope program was closed to the public and accessible only to enrolled AYA via a unique username and password for the duration of the study. Of note, this trial initially began as a pilot to assess the feasibility of multi-site recruitment. Subsequently, it was scaled up to a definitive full-scale clinical trial as a result of increased funding; however, this scale-up occurred without preliminary analysis of pilot outcome data. This trial was registered on ClinicalTrials.gov (NCT02601755) and approved by the respective research ethics board at each study site.

### Participants

Participants were recruited from 5 adult (Nova Scotia Health Authority, Hamilton Health Sciences, Women’s College Hospital, University of Alberta, and Ottawa Hospital) and 5 pediatric (The Hospital for Sick Children [SickKids], IWK Health, Stollery Children’s Hospital, Alberta Children’s Hospital, and the Jim Pattison Children’s Hospital) chronic pain clinics across Canada. Adolescents and young adults were included in the study if they were: (a) between 15–25 years of age, (b) diagnosed with chronic pain of at least 3 months duration according to their medical chart, (c) able to speak and read English, (d) willing and able to complete online measures, (e) actively receiving care at one of the participating pain clinics, (f) on a stable dose of medication for at least 4 weeks prior to entering the study according to their medical chart, (g) able to access an internet-enabled smartphone (iPhone or Android), and (h) able to access an internet-enabled computer or tablet. AYA were excluded if they (a) had significant cognitive impairments as assessed by medical chart review and consultation with participant’s healthcare provider, (b) had major co-morbid illnesses (medical or psychiatric conditions [e.g., conversion disorder, depression, anxiety disorder]) that could impact their ability to understand and use the iCanCope program as determined by their healthcare provider, (c) were from the department of hematology/oncology (only applied to SickKids site), and (d) if they already participated in the earlier iCanCope usability study.

Clinic-based recruitment, both in-person and remotely in response to the COVID-19 pandemic, was used to recruit participants. Potentially eligible participants were identified through clinic appointment lists by the research team. A healthcare provider involved in the patient’s care subsequently assessed capacity and introduced the study when appropriate. Subsequently, the local research team was informed of participant’s interest, and informed consent was obtained. As a result of the COVID-19 pandemic, remote recruitment was adopted whereby eligible participants identified through clinical appointments were introduced to the study via a study information letter (which was mailed/emailed) or during their telemedicine appointment. The research staff scheduled an informed consent discussion to occur over the phone or through a secure video call (Zoom for Healthcare^20^) with the interested potential participants. At telemedicine appointments, the healthcare provider in the circle of care introduced the research team to the interested participant, after which the research staff discussed the study and completed the consent process. In both cases, written consent was obtained via in-person signatures or e-signatures.

### Description of study arms

#### iCanCope intervention

In addition to standard care, AYA randomized into the active intervention received the iCanCope with Pain program for 8 weeks. The iCanCope program was delivered through a mobile app (downloadable from the Google Play [Android] or App Store [Apple iOS]) and website. The app included symptom monitoring, personalized goal setting, library of CBT-based coping strategies, and social support through a community forum feature (further delineated in Supplementary Appendix 1) where participants could interact with other AYA in the study (see Figure 1). Detailed information about the app and its development has been previously reported.^15^ A complementary website provided pain education and self-management information to align with the content on the app. AYA were instructed to log into the app each day and complete the daily symptom tracker. They received a daily push notification at the time of their choice with a reminder to complete the symptom check-in. They were also encouraged to set goals, interact with the library (i.e., read the education content), and engage in the community forum through periodic push notifications; however, these were more passive and less frequent than the daily push notifications for the symptom check-in.

**Figure 1.**
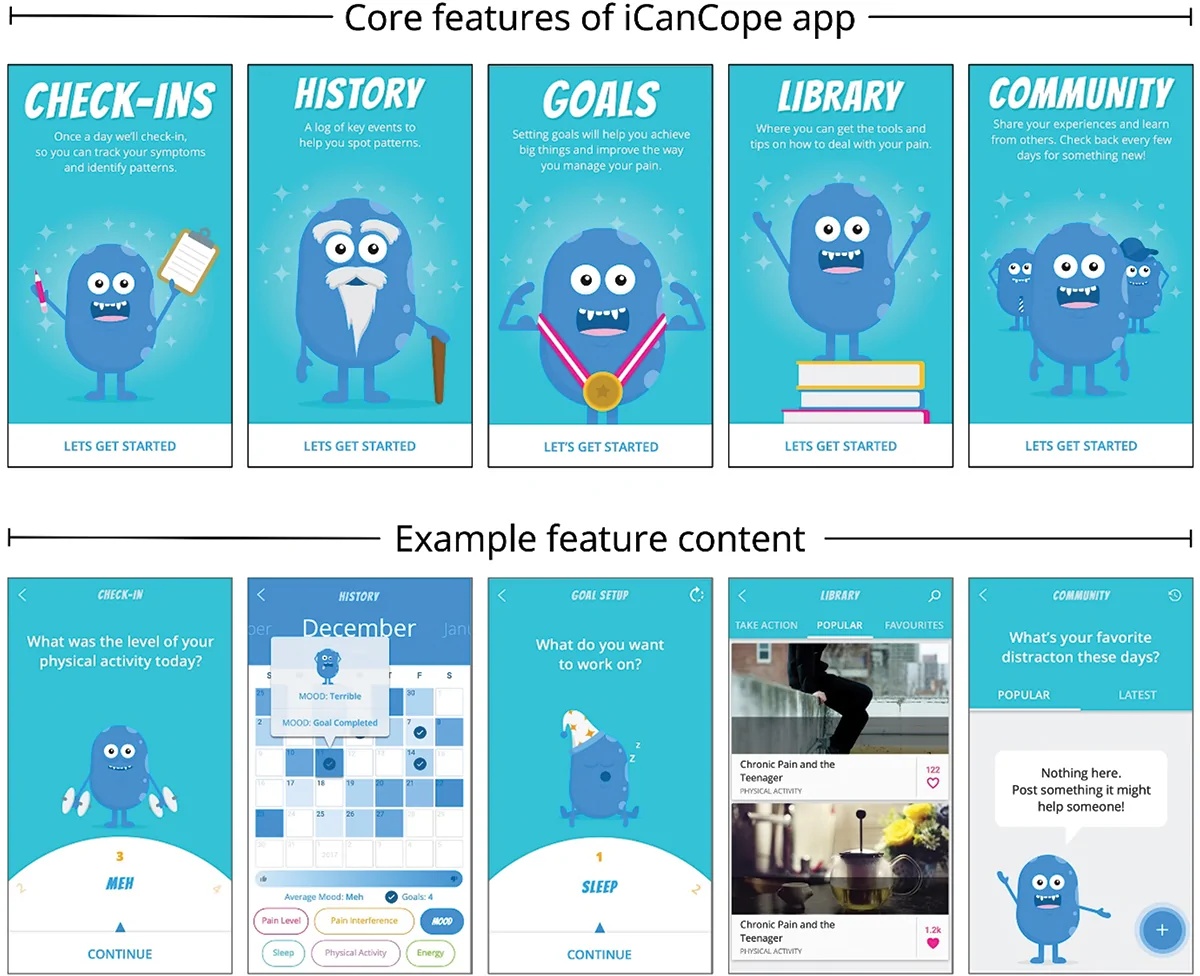
Example screenshots from the iCanCope with Chronic Pain program, including mobile app.

#### Active-control

In addition to standard care, AYA randomized to the active-control group were given access to their own version of the iCanCope app and website. This approach is consistent with recommendations to use reasonable comparison groups rather than no-treatment or usual care.^17^ They received a daily push notification at the time of their choice with a reminder to complete the app symptom check-in and were encouraged to read the education content. The active-control app and website had identical branding to the intervention (e.g., the same colors, fonts, imagery, tone). The active-control app included only the symptom tracking and trending feature, while the website (accessed independently from the app, without ongoing prompts for use) offered basic pain education aligned with the information typically provided by the participating pain clinics.

### Outcomes

All AYA completed self-reported questionnaires at three time points: baseline (T1; after consent, before randomization), after program completion (T2; 8-weeks after randomization), and follow-up (T3; 6 months post‑randomization). Basic demographic, pain characteristics, and treatment expectancy (using an adapted version of the Treatment Expectancy Questionnaire^21^) were collected at baseline to describe the sample. All questionnaires were completed through REDCap, a secure web-based platform which was hosted at SickKids.^22^

#### Implementation and process outcomes

*Accrual and Retention*. Accrual and retention rates were calculated from study tracking logs. Accrual was calculated as those who consented over those approached *and* those randomized over those consented. Retention was measured as those not lost to follow‑up (i.e., those who completed outcome measures) over those randomized. No a priori benchmarks were specified.

*Fidelity*. Fidelity was conceptualized as the successful deployment of the assigned iCanCope app across study arms. Fidelity was measured by the proportion of participants who successfully logged into their assigned app over those randomized. Technical issues related to app deployment or access were also reported. No a priori benchmarks were specified.

*Engagement*. To quantify engagement with the app, compliance with the symptom tracking feature (i.e., completing a check-in each day) was calculated over the 55-day study period for both groups: “low,”≤24% (<13/55 reports); “low-moderate,” 25%–49% (14/55 to 27/55 reports); “high-moderate,” 50%–75% (28/55 to 41/55 reports); “high,” 76%–100% (42/55 to 55/55 reports).^15^ Specific to the intervention group, engagement with the goals, library, and community forum feature was also reported. No a priori benchmarks were specified.

*Acceptability and Satisfaction*. AYA in both study arms completed the Acceptability e-Scale at T2 to measure acceptability and satisfaction with the program, in addition to qualitatively through semi-structured post-study interviews, described below. A sum score of 32 (maximum score 40) on the Acceptability e-Scale was used as the threshold for sufficient acceptability.^23^

#### Efficacy outcomes

The *a priori* primary outcome focused on assessing the efficacy of the iCanCope program as evaluated using the pain interference subscale of the Brief Pain Inventory (summarized as a mean score across 7 items ranging from 0 to 10; higher scores suggest greater pain interference).^24^ Secondary outcomes were: pain intensity (measured using the Brief Pain Inventory^24^), self-efficacy (measured using the Pain Self-Efficacy Questionnaire^25^), emotional wellbeing (measured using the Hospital Anxiety and Depression Scale^26^) sleep function (using the Insomnia Severity Index^27^), social functioning (using the Perceived Social Support from Friends^28^), health-related quality of life (SF-36 Health Survey^29^), patient global impression of change measured (using the Patient Global Impression of Change Scale^30^), and pain knowledge (using a modified version of the Medical Issues, Exercise, Pain and Social Support; MEPS^31^). While health care utilization was also collected for this trial, it was beyond the scope of the present article and as such, was not included. All efficacy outcome measures are described in Table 1.

**Table 1.** Primary and secondary effectiveness measures.

Outcome	Measurement tool	Description
Pain Interference	Brief Pain Invenstory	A 7-item scale used to measure self-reported interference of pain with daily functioning, including general activity, mood, walking ability, normal work, relationships, sleep, and enjoyment of life, among youth with chronic pain. Items are rated on an 11-point numeric rating scale ranging from 0 (“does not interfere”) to 10 (“completely interferes”). Pain interference scores are calculated as the mean of the 7 interference items, with higher scores indicating greater pain-related interference.
Pain Intensity	Brief Pain Invenstory	Self-reported average pain intensity among youth with chronic pain was measured using a single-item. The item was rated on an 11-point numeric rating scale ranging from 0 (“no pain”) to 10 (“pain as bad as you can imagine”). Higher scores indicating greater pain intensity.
Pain Self-efficacy	Pain Self-Efficacy Questionnaire	A 10-item scale used to measure confidence in one’s ability to perform activities and cope with daily life despite pain among youth with chronic pain. Items assess perceived capability to engage in physical activity, social participation, and functional tasks while experiencing pain. Each item is rated on a 7-point Likert scale ranging from 0 (“not at all confident”) to 6 (“completely confident”). Total scores are calculated by summing item responses (range: 0–60), with higher scores indicating greater pain-related self-efficacy.
Anxiety	Hospital Anxiety and Depression Scale	A 7-item scale used to measure symptoms of anxiety among youth with chronic health conditions. Items assess cognitive and emotional aspects of anxiety, including worry, tension, and fear, over the past week. Each item is rated on a 4-point Likert scale (0–3), with total scores ranging from 0 to 21. Higher scores indicate greater anxiety symptom severity.
Depression	Hospital Anxiety and Depression Scale	A 7-item scale used to measure symptoms of depression among youth with chronic health conditions. Items focus on anhedonia, low mood, and reduced enjoyment, excluding somatic symptoms that may overlap with physical illness, over the past week. Each item is rated on a 4-point Likert scale (0–3), with total scores ranging from 0 to 21. Higher scores indicate greater depressive symptom severity.
Insomnia	Insomnia Severity Index	A 7-item scale used to measure the severity of insomnia symptoms among youth with chronic health conditions. Items assess difficulties with sleep onset, sleep maintenance, early morning awakening, satisfaction with sleep, interference with daytime functioning, noticeability of sleep problems, and distress caused by sleep difficulties over the past two weeks. Each item is rated on a 5-point Likert scale ranging from 0 (“no problem”) to 4 (“very severe problem”). Total scores are calculated by summing item responses (range: 0–28), with higher scores indicating greater insomnia severity.
Social Functioning	Perceived Social Support from Friends	A 20-item scale used to measure perceived availability and adequacy of social support from friends among youth with chronic health conditions. Items assess emotional and instrumental support, including feelings of acceptance, understanding, and the ability to rely on friends for help. Items are answered using a dichotomous response format (“yes,” “no,” or “don’t know”), with total scores calculated by summing affirmative responses (range: 0–20). Higher scores indicate greater perceived social support from friends.
Health-related quality of life	SF-36 Health Survey	A 36-item generic health-related quality of life measure used to assess overall physical and mental health functioning among youth and young adults with chronic health conditions. Items are grouped into eight domains: physical functioning, role limitations due to physical health, bodily pain, general health perceptions, vitality, social functioning, role limitations due to emotional problems, and mental health. Domain scores are transformed to a 0–100 scale, with higher scores indicating better health-related quality of life.
Global Impression of Change	The Patient Global Impression of Change Scale	A single-item global rating scale used to measure patients’ perceived change in overall health status since the start of treatment among youth with chronic health conditions. Respondents rate their overall change on a 7-point Likert scale ranging from “very much worse” to “very much improved.” Higher scores indicate greater perceived improvement.
Treatment Expectancy	Treatment Expectancy Questionnaire	A modified version of the Treatment Expectancy Questionnaire was used to assess anticipated benefit of the intervention. Treatment expectancy was measured using a single-item global rating asking adolescents and young adults (AYA): *“How much do you expect the iCanCope educational program will help you manage your chronic pain?”* Responses are rated on an 11-point numeric rating scale ranging from 0 (“don’t think it will help at all”) to 10 (“think it will help a lot”). Higher scores indicate greater perceived treatment expectancy.
Chronic pain knowledge	Medical Issues, Exercise, Pain and Social Support [MEPS] Questionnaire	A modified version of the MEPS was used to assess chronic pain–specific disease and pain management knowledge among adolescents and young adults with chronic pain. The measure consists of 27 items across two subscales: knowledge *about pain* (5 items) and knowledge related to *managing pain* (22 items). Items are rated on a 10-point numeric scale ranging from 1 (“none at all”) to 10 (“know a lot”). No overall summary score is calculated; instead, mean domain scores are derived for each subscale (range: 1–10), with higher scores indicating greater knowledge.

#### Qualitative outcomes

To assess perceptions regarding the value and usefulness of iCanCope as a pain self-management program, a convenience sample of participants from the intervention group completed semi-structured post-study interviews after T2. Audio-recorded interviews were 15–30 minutes in duration and conducted over the telephone.

### Sample size and power considerations

Based on Hertzog’s^32^ recommendations of 20–30 participants per group for pilot studies, the initial pilot trial aimed to recruit a total of 120 participants (i.e., 20 adolescents and 20 young adults per experimental and control group, stratified by age [i.e., adolescents (15–17 year olds) and young adults (18+)]). Upon extension to the full-scale RCT, the sample size was determined based on the anticipated effect size of Cohen’s d = 0.25 based on prior literature for the main outcome of pain intensity assuming 80% power and a two-sided alpha of 0.05 and 30–40% refusal rate based on previous experience in this population, resulting in an additional 180 participants to achieve a total sample size of 300 participants.^33^

### Randomization

A secure, web-based randomization service (www.randomize.net) was used to allocate participants to trial groups and ensure hidden allocation. The allocation process was balanced by study center to control for center‑specific disease education, treatment approaches, and age. The randomization process occurred following informed consent and baseline measurement completion, after which the lead center’s (*SickKids*) study coordinator completed randomization to determine group allocation.

### Blinding

This was a single‑blind study whereby the study participants were not explicitly told which group they were assigned to. The research team completed a guided, standardized orientation over a secure video call (Skype or Zoom for Healthcare^20^) or telephone with each participant, from both the intervention and active-control arms. Both study arms were objectively described to participants (excluding which was the intervention and which was the active control) and both received access to a version of the iCanCope application. The orientation also included instructions on how to download their assigned iCanCope app, login credentials (unique username and password), complete their first check-in and set check-in reminders (for those in the intervention arm, it also included going through the additional features on the app) and website on their personal devices and confirmed sign-in fidelity with their unique login credentials. Both versions of the program were identically branded (i.e., same font, colors, tone). This approach was intended to preserve treatment credibility (believability of treatment) and expectancy (belief in personal benefits). Individual‑level analytics were captured from the app through APEEE (Analytics Platform to Evaluate Effective Engagement), a validated and secure platform to assess user engagement with digital health programs.^34^

### Analysis

#### Quantitative analysis

Demographic, disease characteristics, pain management, and treatment adherence were described with mean and standard deviation for continuous variables and raw counts and percentages for categorical variables by allocation group. An intent‑to-treat approach was used where all participants were included in the final analysis according to the allocation group to which they were randomized. To assess the efficacy of the iCanCope program on primary and secondary outcomes, linear mixed models using maximum likelihood estimation were used with a binary indicator for allocation group, 3-level indicator for assessment time points and interaction of group and time as fixed effects and intercepts for each participant as random effects. A significance level of 0.05 for the primary outcome and a Bonferroni-adjusted alpha level of 0.0031 was used to maintain an overall level of 0.05 for all secondary outcomes. Missing outcome data were assumed to be missing at random as supported by comparisons of baseline characteristics between participants with complete and incomplete follow-up data (see Supplementary Appendix 1; Supplementary Tables 1).

Two sensitivity analyses were completed. First, participants initially recruited in the pilot trial and those who completed the full-scale trial were compared descriptively, and second, a per-protocol analysis was completed. Finally, two exploratory post hoc analysis was performed. First, to understand the impact of treatment expectancy, which previous research suggests is predictive of patient outcomes and moderates treatment response in clinical trials.^35^ Second, given the wide age range of participants included in this study that captured two developmental periods, the moderating effect of age on pain intensity and pain interference was assessed. In addition, there is growing recognition on comorbid sleep disorders in AYA with pain and its potential impact on treatment response, to understand this we assessed the moderating effect of insomnia on pain intensity and pain interference.^36–38^ These analyses are detailed in Supplementary Appendix 1. Data analysis was completed using STATA version 15.1 and R version 4.2.0.^39,40^

#### Qualitative analysis

Participants in the intervention group were invited to take part in an optional individual interview to share their experiences using the program. All interviews were audio-recorded, transcribed, and deidentified and were conducted by trained research coordinators from the main study site. Interview transcripts underwent descriptive content analysis, as described by Sandelowski,^41^ using Dedoose *9.0.17*.^42^ Multiple coders (CL; VM; BM; RK) independently reviewed transcripts and met collaboratively to reflexively discuss and iteratively develop an inductive codebook. The analytic team considered how researchers’ perspectives, including the lived experience of chronic pain among some members, may shape interpretation. A portion of transcripts (25%) was double-coded and reviewed by all analysts to ensure consistency and agreement for code application, with discrepancies resolved through discussion until consensus was reached. Analytic decisions were documented throughout the coding process. Finally, authors CL and FN synthesized the coded data to summarize the key sentiments regarding user experience.

## Results

### Accrual and retention

Participants were enrolled from February 2017 to December 2021 (when the sample size was achieved), with the final follow‑up completed in September 2022. A convenience sample of 349 participants was consented to the study, of which 302 were randomized (150 intervention; 152 control). The Consolidated Standards of Reporting Trials (CONSORT) diagram is presented in Figure 2 which presents the screening and eligibility data. The final analyzed sample included 297 participants; five participants were removed because they did not complete baseline questionnaires, as such the final sample included 147 participants in the intervention and 150 in the control group. Since this trial started as a pilot, the first 72 participants (33 intervention; 39 control) were consented to complete only T1 and T2; the remaining participants completed the full study protocol which included the follow‑up time point. The accrual rate was 64% (349/541; those consented over those approached) and 87% (302/349; those randomized over those consented). The retention rate at T2 was 89% (131/147) for the intervention group and 92% (138/150) for the control group; at T3, it was 71% (81/114) and 72% (80/111) respectively. No adverse events were reported by participants over the duration of the project.

**Figure 2.**
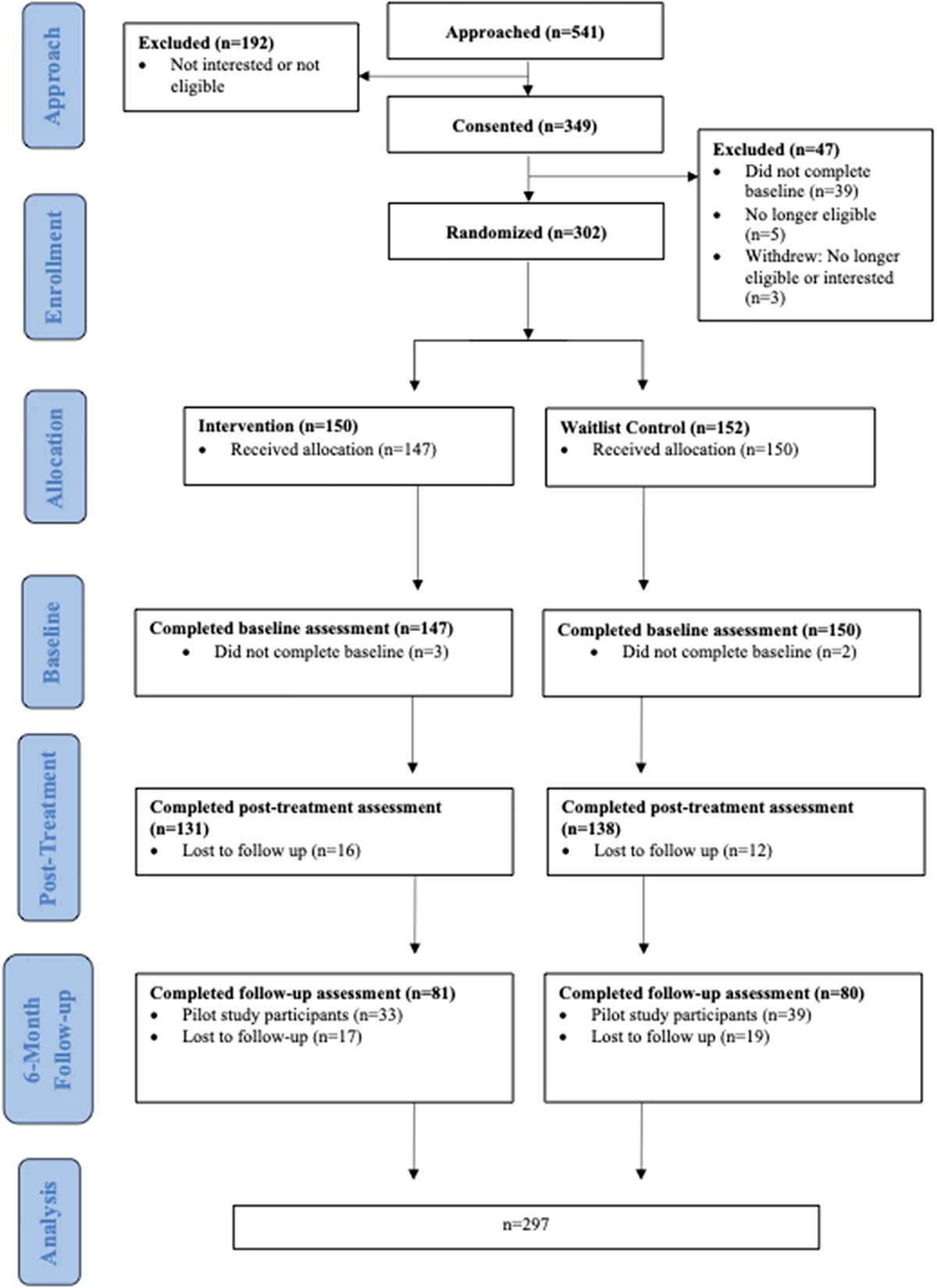
Consolidated standards of reporting trials flow diagram.

### Fidelity

Fidelity was high across study arms, with 93% (140/150) of participants in the intervention group and 91% (138/152) in the control group successfully logging into their assigned app. No technical issues were documented that affected delivery of the app.

### Participant characteristics

Table 2 reports participant demographics and baseline characteristics. Most participants were female (85%, 252/297), identified as White (73%, 218/297), and were aged 15–25 years with a median age of 17 (Interquartile Range [IQR] 15–18). While the distribution of pain location varied, it was similar across groups, with a median of 2 pain sites (IQR 1–3) and median pain duration of 4 years (IQR 2.25–7). Most AYA were actively receiving multidimensional pain management from their respective pain clinics, when categorized using the 3P (physical, psychological, and pharmacological) approach (Figure 3). No meaningful differences in demographic or clinical characteristics were identified between participants who were recruited for the pilot compared the full-scale RCT (Supplementary Table 2).

**Table 2.** Adolescent and young adult demographics and pain characteristics.

	Intervention (n = 147)	Control (n = 150)
Age in years, median (IQR)	17 (15–17)	17 (16–18)
Gender, n(%)		
*Female*	127 (89%)	125 (86%)
*Male*	14 (9%)	20 (14%)
*Non-binary*	1 (0.7%)	1 (0.6%)
*Missing*	5	3
Race, n(%)		
*Indigenous*	1 (0.7%)	2 (3%)
*Arab/West Asian*	3 (2%)	1 (0.7%)
*Asian (Chinese, Filipino, Korean, South Asian, South East Asian)*	5 (3%)	12 (8%)
*Black*	2 (1%)	2 (1%)
*Latin American*	1 (0.7%)	1 (0.7%)
*White*	112 (79%)	106 (75%)
*Mixed Race*	14 (9%)	12 (8%)
*Other*	3 (2%)	4 (3%)
*Missing*	6	8
Location of Pain^a^		
*Abdominal*	41 (28%)	47 (32%)
*Facial*	15 (10%)	12 (8%)
*Headache*	58 (39%)	63 (42%)
*Low back*	60 (41%)	61 (41%)
*Musculoskeletal*	55 (37%)	60 (40%)
*Neuropathic*	35 (24%)	35 (23%)
*Pelvic*	19 (13%)	35 (23%)
*Other*	48 (33%)	40 (27%)
Number of Pain Sites, median (IQR)	2 (1–3)	2 (1–3)
Duration of pain in years^b^, median (IQR)	4 (2.5–7)	4 (2–7)

^a^Participants were able to select multiple pain areas.

^b^Missing values for duration of pain duration (N = 7), control (N = 9).

**Figure 3.**
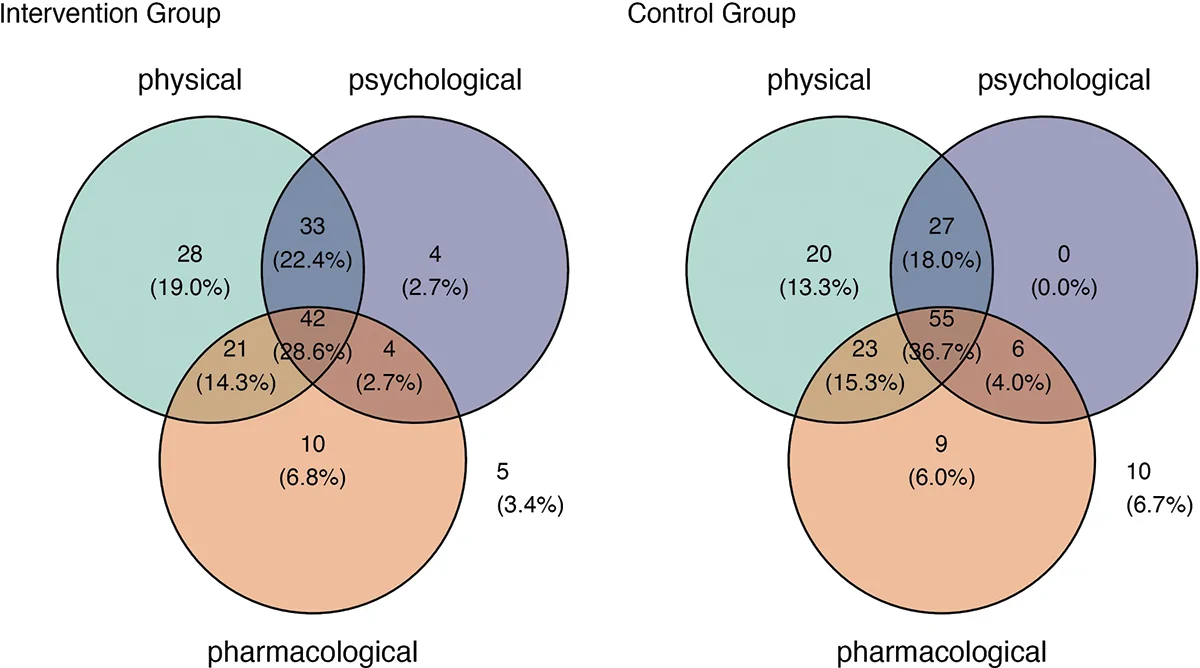
Baseline use of psychological, Physical Therapy, and pharmacological pain management.

### Engagement with the iCanCope program

Check-in compliance was high, with most AYA, regardless of group, falling in the high-moderate or high check-in category (see Supplementary Figure 1). AYA in the intervention group also engaged with the additional features of the app: with 76% completing one goal (median goals 3 [IQR: 2–7.5]), 72% reading an article (median 4 [IQR: 2–8]), and 93% viewing the community forum. Specific to the community forum, 52% of the intervention group posted on the forum (median number of posts 3 [IQR: 2–7] and 43% liked posts on the forum (median number of likes 3 [IQR: 1–5]).

### Treatment efficacy

Means and standard deviations for all efficacy outcomes are presented in Supplementary Table 3. For the primary outcome of pain interference, use of the iCanCope program did not result in significant differences compared to the control version of the app (β = −0.40, 95% CI: −0.88 to 0.84; p = .10). There was, however, a significant time effect, with AYA in both groups showing reductions in pain interference over the study period (Table 3). For secondary outcomes, no significant treatment effects were observed. Improvements over time were noted for the SF-36 Pain domain and the “About Pain” and “Managing Pain” subscales of the modified MEPs, suggesting overall increases in pain knowledge and reductions in pain regardless of group allocation (Table 3). In the per-protocol analysis, nine participants from the intervention group were dropped due to non-engagement with the app. Results were unchanged from the primary analysis (see Supplementary Table 4). Visual inspection of residual plots revealed no obvious deviations from homoscedasticity or normality.

**Table 3.** Adolescent and young adults reported primary and secondary outcomes, linear mixed models.

Outcome	Predictor	Β (95% CI)	p-value
Pain Interference	Intervention	−0.40 (−0.89, 0.08)	0.10
	**Time 2**	**−0.58 (−0.90, −0.26)**	**0.0004**
	**Time 3**	**−0.64 (−1.04, −0.24)**	**0.001**
	Intervention x Time 2	0.07 (−0.39, 0.53)	0.76
	Intervention x Time 3	0.01 (−0.55, 0.57)	0.96
Average Pain Intensity	Intervention	−0.10 (−0.50, 0.29)	0.61
	Time 2	−0.14 (−0.41, 0.13)	0.31
	Time 3	−0.20 (−0.53, 0.13)	0.23
	Intervention x Time 2	−0.16 (−0.55, 0.22)	0.39
	Intervention x Time 3	−0.53 (−0.99, −0.06)	0.03
Pain Self-Efficacy	Intervention	2.58 (−0.32, 5.47)	0.08
	Time 2	1.18 (−0.71, 3.07)	0.22
	Time 3	2.03 (−0.29, 4.35)	0.08
	Intervention x Time 2	0.99 (−1.71, 3.70)	0.47
	Intervention x Time 3	−0.77 (−4.05, 2.51)	0.64
Social Support	Intervention	−0.28 (−1.50, 0.93)	0.65
	Time 2	−0.24 (−0.97, 0.49)	0.53
	Time 3	0.22 (−0.69, 1.12)	0.64
	Intervention x Time 2	0.17 (−0.88, 1.22)	0.75
	Intervention x Time 3	−0.53 (−1.81, 0.75)	0.42
Anxiety	Intervention	−0.21 (−0.89, 0.46)	0.54
	Time 2	−0.05 (−0.59, 0.48)	0.85
	Time 3	0.13 (−0.52, 0.78)	0.69
	Intervention x Time 2	−0.09 (−0.85, 0.67)	0.81
	Intervention x Time 3	−0.38 (−1.29, 0.54)	0.42
Depression	Intervention	−0.11 (−0.69, 0.48)	0.71
	Time 2	0.18 (−0.26, 0.62)	0.42
	Time 3	0.27 (−0.26, 0.81)	0.32
	Intervention x Time 2	−0.08 (−0.71, 0.55)	0.80
	Intervention x Time 3	−0.43 (−1.19, 0.33)	0.26
Insomnia	Intervention	0.31 (−1.18, 1.81)	0.68
	Time 2	−0.19 (−1.13, 0.74)	0.68
	Time 3	−0.76 (−1.92, 0.39)	0.19
	Intervention x Time 2	−0.49 (−1.84, 0.85)	0.47
	Intervention x Time 3	−0.81 (−2.44, 0.82)	0.33
SF-36 Physical Functioning Domain	Intervention	3.48 (−2.32, 9.28)	0.24
	Time 2	4.21 (1.06, 7.35)	0.008
	Time 3	4.65 (0.77, 8.54)	0.02
	Intervention x Time 2	−0.42 (−4.93, 4.08)	0.85
	Intervention x Time 3	−1.78 (−7.30, 3.73)	0.52
SF-36 Physical Limitations Domain	Intervention	3.99 (−4.31, 12.29)	0.34
	Time 2	3.45 (−3.13, 10.03)	0.30
	Time 3	3.23 (−4.78, 11.23)	0.43
	Intervention x Time 2	6.50 (−2.94, 15.94)	0.18
	Intervention x Time 3	5.61 (−5.77, 16.99)	0.33
SF-36 Limitations of Emotional Health Domain	Intervention	0.30 (−8.95, 9.56)	0.95
	Time 2	−1.99 (−8.66, 4.66)	0.56
	Time 3	−0.68 (−8.83, 7.46)	0.87
	Intervention x Time 2	3.22 (−6.34, 12.79)	0.51
	Intervention x Time 3	6.53 (−5.05, 18.11)	0.27
SF-36 Energy and Fatigue Domain	Intervention	1.39 (−3.45, 6.23)	0.57
	Time 2	1.72 (−1.09, 4.52)	0.23
	Time 3	1.22 (−2.22, 4.67)	0.48
	Intervention x Time 2	1.03 (−2.99, 5.06)	0.61
	Intervention x Time 3	2.77 (−2.13, 7.67)	0.27
SF-36 Emotional Wellbeing Domain	Intervention	1.06 (−3.13, 5.25)	0.62
	Time 2	−0.39 (−3.12, 2.33)	0.78
	Time 3	0.40 (−2.94, 3.75)	0.81
	Intervention x Time 2	−1.88 (−5.80, 2.03)	0.34
	Intervention x Time 3	−1.67 (−6.42, 3.08)	0.49
SF-36 Social Functioning Domain	Intervention	2.80 (−3.19, 8.80)	0.36
	Time 2	3.55 (−0.63, 7.73)	0.10
	Time 3	3.39 (−1.74, 8.51)	0.19
	Intervention x Time 2	−0.58 (−6.59, 5.43)	0.85
	Intervention x Time 3	0.61 (−6.67, 7.89)	0.87
SF-36 Pain Domain	Intervention	3.89 (−0.42, 8.22)	0.08
	**Time 2**	**5.19 (1.85, 8.53)**	**p < .003**
	**Time 3**	**6.55 (2.49, 10.60)**	**p < .003**
	Intervention x Time 2	−0.61 (−5.39, 4.17,	0.80
	Intervention x Time 3	−0.03 (−5.79, 5.73)	0.99
SF-36 General Health Domain	Intervention	−0.04 (−4.05, 3.97)	0.98
	Time 2	0.14 (−1.85, 2.13)	0.88
	Time 3	1.85 (−0.61, 4.32)	0.14
	Intervention x Time 2	2.26 (−0.60, 5.12)	0.12
	Intervention x Time 3	0.86 (−2.64, 4.36)	0.63
Pain Knowledge – About Pain Domain	Intervention	−0.27 (−0.77, 0.22)	0.28
	Time 2	0.39 (0.08, 0.69)	0.01
	**Time 3**	**0.98 (0.62, 1.35**	**p < .003**
	Intervention x Time 2	0.34 (−0.08, 0.77)	0.11
	Intervention x Time 3	0.29 (−0.23, 0.81)	0.27
Pain Knowledge – Managing Pain Domain	Intervention	−0.18 (−0.60, 0.24)	0.39
	**Time 2**	**0.54 (0.28, 0.81)**	**p < .003**
	**Time 3**	**1.05 (0.72, 1.37)**	**p < .003**
	Intervention x Time 2	−0.01 (−0.39, 0.37)	0.96
	Intervention x Time 3	0.02 (−0.44, 0.48)	0.93

Note: Bolded values indicate statistical significance at an alpha level of 0.05 for the primary outcome (pain interference) and a Bonferroni-adjusted alpha of 0.0031 for secondary outcomes.

#### Post-hoc analysis – treatment expectancy

Treatment expectancy (TE) was slightly higher in the intervention group reporting a mean score of 6.74 (SD 2.06) compared to the control 5.93 (SD 1.9). Controlling for pre-intervention TE did not result in a meaningfully different beta coefficient for the intervention effect on pain interference (the primary outcome), nor did it change the number of secondary outcomes with a significant treatment effect. The main effect of TE was insignificant for both primary and secondary outcomes at T2 and T3. Regression outputs are presented in Supplementary Table 5. This suggests that controlling for TE did not enhance the ability to detect intervention efficacy or effect outcome improvements over time.

#### Post-hoc analysis – moderating effect of age and insomnia

Age was not found to moderate the intervention effects (Supplementary Table 6); however, a significant three-way interaction was observed for the moderating effect of insomnia on pain interference (β = –2.06, p = .000) and pain intensity (β = –1.01, p = .038) at 6 months, suggesting that the intervention’s effect over time may differ depending on baseline insomnia status (see Figure 4; Supplementary Table 6).

**Figure 4.**
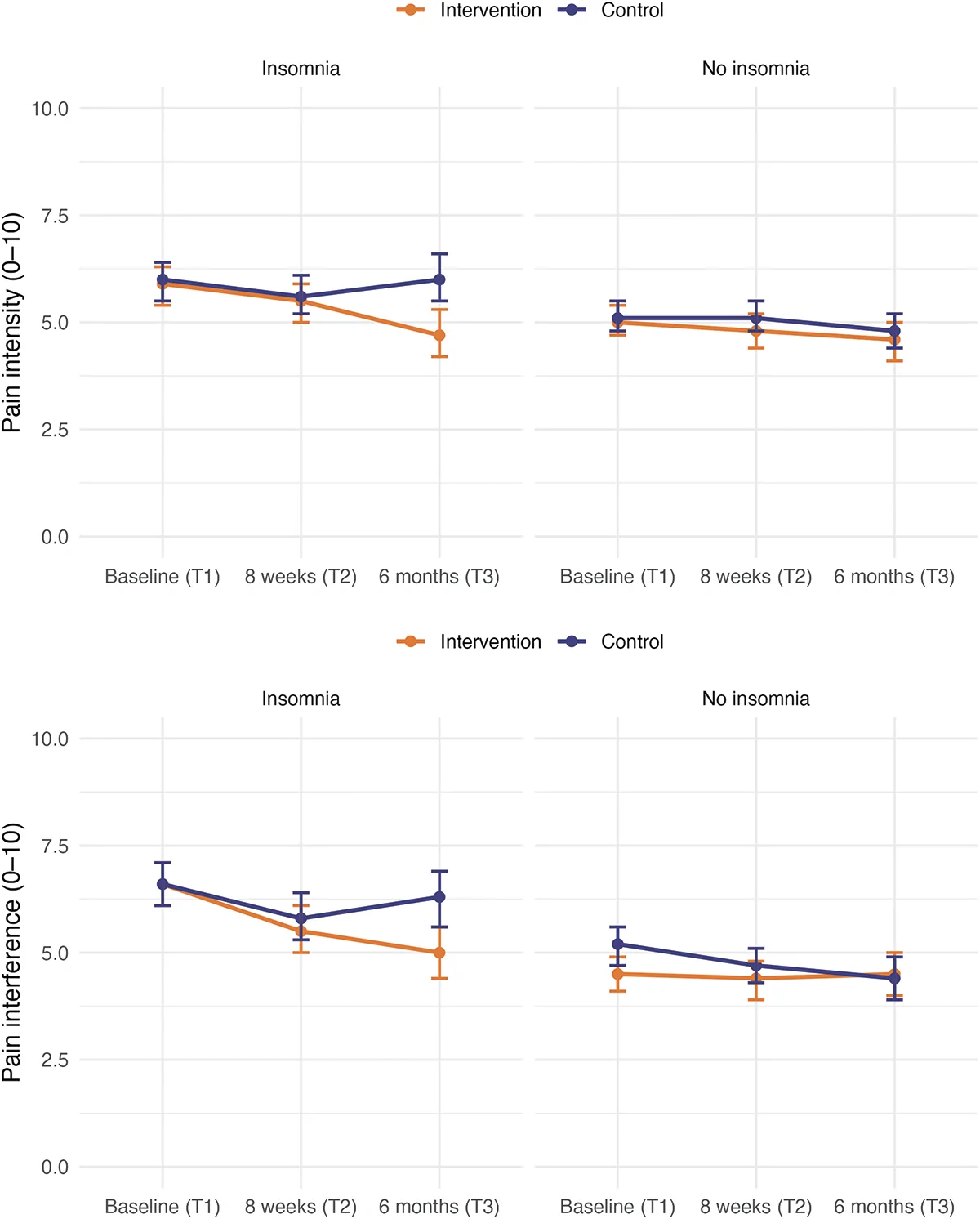
Estimated mean pain intensity and pain interference over time by intervention group and insomnia status.

### Acceptability and satisfaction

#### Quantitative

Acceptability measured with the app was high in both the intervention (33.9 ± 7.0) and control group (32.7 ± 6.8). Two-thirds of participants were also interested in using the app long-term, with 65.1% (intervention) and 66.6% (control) interested in using it for ≥8weeks. In comparison, the website received lower scores of acceptability and satisfaction, with intervention AYA rating it 25.9 ± 9.2 while control AYA found it 25.8 ± 8.7. Furthermore, most AYA were not interested in using the website long term with 29.4% (intervention) and 33.0% (control) interested in using it for ≥8weeks.

#### Qualitative

A total of 16 individual interviews were conducted with intervention group participants. The interviewees ranged in age from 15 to 24 years (mean 16.6; SD 2.2) and nearly all identified as being female (94%). Program engagement, as per completion of daily symptom check-ins over the study period, ranged from low-moderate (2; 12%) to high-moderate (7; 44%) to high (7; 44%).

*How Would AYA Describe iCanCope to Peers with Chronic Pain*. When asked how they would describe the iCanCope program to other AYA living with chronic pain, all respondents portrayed the program in a positive manner. Participants emphasized program simplicity and convenience, such as: *“A very easy like not complicated app … if they have like a very busy day and they just wanted to do something simple every day to kind of check in”* (Participant 067, 15-year-old female). They highlighted the core feature of tracking pain and function, explaining that:

“It’s a really good way to see not only like my pain fluctuations, but how everything like my sleep, my mood and everything around that kind of affects that, like in a day. And so I can like take preventative action to help me, you know, cope with that a little bit better” – Participant 012, 16-year-old female

Others described it as a way to stay organized and prepare for medical appointments, noting *“it was helpful for me because I know that for every doctor’s appointment I go to I need to remember how my pain was … really helpful for just keeping track of it in one place”* (Participant 014, 15-year-old, female). Interviewees also highlighted the value of goal-setting and community forum features: *“I feel like making goals … it’s another step to kind of take control over your chronic pain. And if you’re someone who has some trouble with that, then I think that tool is very, very helpful.”* (Participant 052, 15-year-old, female); *“… you can go learn tips and interact with other people”* (Participant 017, 17-year-old, female).

In terms of user experience, the app was portrayed as fun and empowering, making pain self-management a less clinical experience:

“It basically is like a fun way to get kids to … interact with, like their pain and their medical stuff, because like most kids don’t want to do anything for that, just like give them the medication and leave. So [I would describe it] like a fun little way to get kids to interact and start learning about their pain and like kind of taking it on as their own thing too.” – Participant 104, 16-year-old, female

Overall, iCanCope was framed as a valuable resource to support healthy coping: “I think the program is about learning how to deal, in a healthy way, with your illnesses and the problems that come up with them” – Participant A006, 24-year-old, female.

*Program Design*. Interviewees consistently described the program design as inviting, clear, and fun, emphasizing the value of AYA-friendly graphics and colors: *“bright, fun, less scary, not really like pain, just something fun”* (Participant 061, 16-year-old, female). The Copey character was characterized as likeable and useful: “*little characters … made the app more unique*” (Participant 013, 16-year-old, female) and *“different expressions on [Copey’s] face … help you identify instead of just having a 1 to 10 … you can look at his face and think, ‘oh I kind of felt like that today.’ I think the character really helped and … it would be appropriate for anyone”* (Participant 019, 17-year-old, female). The “spinning wheel” design for data input was reported to be intuitive and enjoyable: “*so easy, so fun … just spin it … fast and easy*” (Participant 009, 17-year-old, female). Data visualizations such as the heatmap calendar for viewing symptom trends were valued for encouraging pattern recognition: “*bright vs faded blues … clearly see which day I did better on*” (Participant 037, 15-year-old, female). Participants also identified opportunities for improvement such as adding new parameters to the symptom check-in: “*stress isn’t one of the questions … hard to put on a scale*” (Participant 005, 17-year-old, female). Others felt that the aesthetic was *“targeted towards younger adolescents”* (Participant 061, 16-year-old, female), suggesting the addition of age-based avatars. Overall, interviewees perceived the program design as approachable and encouraging: *“it’s like actually cute and fun … something you actually want to do … I think the best thing about it was it just wasn’t like all formal medical.”* (Participant 104, 16-year-old, female).

*Perceptions about the Symptom Check-in and History*. AYA reflections on the program often highlighted the standalone value of the check-in and history features. For instance, some interviewees described the check-in as *“the main part”* they used and often their first action on opening the app: *“most often I would just use the check-in”* (Participant 024, 17-year-old, female) and *“when I open the app I’d go to check-in first”* (Participant A006, 24-year-old, female). The feature’s simplicity and speed were highlighted: *“it was just a very simple brief questionnaire essentially, but then at the end pretty much each day you get information to help you”* (Participant 067, 15-year-old, female); *“better than just entering numbers … fun to spin and see avatars”*(Participant 014, 15-year-old, female). Check-in notifications also supported routine formation: *“without the notifications, I would have missed a lot more days than I did”* (Participant 013, 16-year-old, female) and some users anchored app use to consistent times: *“I would do it every night at 8 pm [when I took my meds]”* (Participant 024, 17-year-old, female).

Interviewees also reported reflection and self-awareness benefits of the check-in and history: *“it was almost therapeutic to reflect back on my day”* (Participant 037, 15-year-old, female); *“I found the sleep [question] helped me a lot because it made me realize if my pain is getting worse if I get a certain amount of sleep or if my sleep is really off that night and the energy levels too because I can either see if the pain was getting worse if I was more awake or if I was more exhausted that day.”* (Participant 013, 16-year-old, female). The history feature amplified this value by enabling trend tracking and real world application: *“I could go to the calendar and see everyday … how I was feeling compared to the last one and kind of track my progress”* (Participant 037, 15-year-old, female) and supported clinical recall: *“I went back to it to tell the doctors when the flare-up started and I found that useful, too”* (Participant 104, 16-year-old, female).

*Reflections on Program Impacts*. Most (15/17) interviewees reported that using iCanCope enhanced their confidence and/or ability to engage in pain self-management. One participant explained, *“I got better knowledge of my pain cycle … learning stuff in the articles about sleep and tips on being healthy”* (Participant 009, 17-year-old, female), while another noted, *“the sleep ones helped me a lot because it made me realize my pain is getting worse if I get a certain amount of sleep”* (Participant 013, 16-year-old, female). For some participants, this greater awareness translated into behavioral changes and problem-solving, as AYA described using insights to adjust their routines: *“Realized I had pain when I did this … maybe next time I can manage my pain better”* (Participant 037, 15-year-old, female); “*seeing patterns … how everything like my sleep, my mood and everything around that kind of affects that … so I can take preventative action to help me cope better* ” (Participant 012, 16-year-old, female). Goal-setting features were particularly motivating, fostering a sense of accomplishment: “*Goal setting gave me a sense of accomplishment … I’m managing my pain enough to accomplish this goal*” (Participant 037, 15-year-old, female).

Over time, some participants described a shift toward feeling ownership over their disease management: *“From ‘this is for somebody else’ to ‘I want to do this’”* (Participant 061, 16-year-old, female). This same participant reflected: “*it actually really like surprised me how much it helped. And I notice that my pain did get better over time and I don’t know if I would’ve noticed it otherwise” –* Participant 061, 16-year-old, female. Similarly, another participant shared: *“… it gave me a chance to actually stop and like re-evaluate, ‘how am I doing?’”* (Participant 052, 15-year-old, female).

Beyond pain management, the app also supported broader personal goals, such as overcoming social anxiety. For instance, one participant noted that tracking goals encouraged them in *“doing things that made me a little bit uncomfortable that I still wanted to do”* (Participant 012, 16-year-old, female), such as joining a school play and making new friends. Participants also highlighted that the program promoted mindfulness and self-awareness: *“just in general, [the app] just made me pay attention to myself more than just like constantly doing stuff and working”* (Participant 104, 16-year-old, female); *“it asked me what I thought I could do to improve … that was good cause then I thought, ‘oh what can I do more easily?,’ do some stuff to feel better”* (Participant 014, 15-year-old, female).

*Motivators and Barriers to Program Use*. Motivating factors identified by participants included program convenience and ease of use: *“it never took very long … I’d go on, do the quick questions and then get off”* (Participant 102, 15-year-old, male) and *“I liked how easy it was to use and set up … notifications make you wanna use the app more”* (Participant 013, 16-year-old, female). Goal setting and feedback were also strong motivators: *“it was a good way to kind of track progress, like it was right in front of your face”* (Participant 061, 16-year-old, female); *“[feedback from app] helped me improve what I’m doing in my day and how to manage my pain”* (Participant 037, 15-year-old, female). The availability of curated educational resources also played a motivating role: *“instead of going straight to Google, I could go to this and it had everything I needed specifically”* (Participant 017, 17-year-old, female). Overall, participants characterized the program content as credible and reassuring: *“it was a good place to get trustworthy advice and keep all that information together”* (Participant 014, 15-year-old, female); *“I like how its private like especially when it was asking us questions in the community section … I liked how we had our own little like social media app for people with pain”* (Participant 017, 17-year-old, female).

Barriers to program use included perceptions of unachievable suggestions: *“some of it [is] really accurate, [but] … trying to get 9 hours of sleep … that’s not really achievable. I almost feel like it could kind of make people feel unmotivated some of it, just because it was these super high standards like a perfect thing like some people wouldn’t be able to manage”* (Participant 061, 16-year-old, female), forgetting to use the program (*“I forgot to do it quite a few times … but I know that I did it at least every second day”* (Participant 061, 16-year-old, female), and a desire for additional features: *“if medication [tracking was added], it would just help even more and drive me to do it every day”* (Participant 067, 15-year-old, female).

## Discussion

Chronic pain in adolescents and young adults is increasingly common and, when inadequately managed, can have long-term consequences for health-related quality of life. Digital technologies offer an opportunity to deliver chronic pain education and psychosocial support through accessible and scalable platforms. The current study evaluated the effectiveness of the iCanCope program among AYA with chronic pain. While no statistically significant improvements were observed in pain or other clinical outcomes, the study provides important insights into engagement, acceptability, and user experience which were rated highly.

### Comparison to existing literature

Internet-delivered and smartphone-based interventions for individuals with chronic pain have been evaluated across a range of populations, with mixed findings reflecting heterogeneity in study designs, control conditions, and samples. The iCanCope program specifically has been assessed in several clinical populations, also with mixed results.^43–45^ Among youth with sickle cell disease, treatment effects on pain were observed; however, this study used an active education control group that received access to a website rather than a modified version of the iCanCope app as used in the present study.^43^ A pilot trial in youth with juvenile idiopathic arthritis, which employed an active control condition (i.e., modified version of the iCanCope app with only symptom tracking) similar to the present study, found high iCanCope app engagement across both study groups but no treatment effects.^44^ Notably, both arms demonstrated clinically meaningful reductions in arthritis pain intensity over time, a pattern that was not observed in the present study.^44^ A single study in adults with Neurofibromatosis Type 1 reported improvements in pain among iCanCope app users; however, this trial employed a unique three-arm design that included iCanCope with contingency management (i.e., financial incentives for engagement).^45^ Direct comparability with the present study is therefore limited due to differences in study design and the adult sample.

More broadly, most Internet-delivered cognitive behavioral therapy interventions have demonstrated improvements in pain outcomes or at least one other clinical outcome, such as sleep or activity limitations.^33,46–48^ Importantly, these studies predominantly used non-active control conditions, including waitlist or pain education–only controls. Similarly, a single smartphone-based intervention evaluated using a cluster randomized controlled trial with schools as the unit of randomization found improvements in emotional, social, and school functioning, but not in pain outcomes.^49^ In contrast, a Cochrane review of psychological therapies for mixed chronic pain conditions found no beneficial effect on pain intensity at post-treatment or follow-up, although the quality of the evidence was rated as low.^50^

Overall, the existing literature suggests that studies using waitlist or education-only controls tend to demonstrate clearer intervention effects, whereas those employing active control conditions report more modest or null intervention effects. Secondly, clearer results tend to emerge in studies where pain is attributable to a primary disease (e.g., sickle cell disease, juvenile idiopathic arthritis), compared to a primary chronic pain diagnosis.^43,44^ The current study contributes to this body of evidence by evaluating the iCanCope intervention using a more rigorous active control design (i.e., modified version of the iCanCope app with only symptom tracking compared to version of the app with symptom tracked and additional features [goal setting, library, and community forum]) in an AYA chronic pain population that is generally understudied.^14^

### Interpretation of lack of treatment effects

Several factors may help explain the absence of observed intervention effects. First, in line with recommendations for digital health trials, the current study employed an active control by offering the iCanCope app without additional features (goal setting, library, and community forum) to AYA in the control group. While methodologically rigorous, this design likely minimized detectable differences between groups, in contrast to many digital health intervention studies that rely on waitlist or education-only control conditions.^17–19^ Second, participants in both groups demonstrated high engagement with the shared check-in feature, while use of the additional intervention features was more moderate. Qualitative findings suggest that symptom tracking through the check-in feature was experienced as reflective, meaningful, and supportive, which may explain improvements over time in both groups and further reduce the between-group contrast. In contrast, the comparatively lower engagement with more active or behaviorally demanding features (e.g., goal-setting, community forum, or library) may indicate barriers related to usability, perceived relevance, or the level of effort required. This suggests that the additional features may require optimization or expansion to produce detectable intervention effects. For example, it’s possible the structured and prompt-based nature of the community forum may have hindered meaningful peer interaction, as it did not foster a true sense of community. A better balance between moderation and fostering community should be achieved. Third, although participants reported moderate pain severity and some impairment at baseline, many also reported functioning well in certain psychosocial domains (i.e., social functioning, emotional wellbeing, pain-self efficacy). Moreover, many participants were already receiving multidimensional pain management and a high level of specialized care given the recruitment setting (i.e., urban academic teaching hospitals), which may have limited the potential for additional benefit from the iCanCope intervention. It may be that iCanCope is better suited at a different point in the pain management trajectory, potentially for AYA awaiting access to tertiary pain services, those transitioning out of tertiary care, or those self-managing their pain in the community. Future research should explore the effectiveness of iCanCope among AYA who don’t have access to high intensity pain care. Fourth, the present study found no moderating effect of age on pain intensity or pain interference. Given that most AYA were adolescents (15–17 years), and those categorized as young adults were primarily 18–19 years old, this may reflect limited variability in developmental stage within the sample. As such, differences in engagement with the digital health intervention and self-management capacity may not have been sufficiently distinct to influence the results. Fifth, while AYA qualitatively described improvements in social participation, autonomy, and self-reflection, these constructs were not explicitly measured as study outcomes. Collectively, these factors likely reduced the ability to detect intervention effects in this trial. However, the absence of detectable clinical effects does not necessarily indicate a lack of benefit, as AYA described meaningful and supportive experiences in qualitative findings.

### Implications

The study findings offer important insights into how AYA engage with and perceive digital pain self-management tools. High engagement and acceptability, alongside positive qualitative feedback, underscore the relevance and usability of the iCanCope program in this population. Specifically, the check-in feature emerged as a particularly valued component of the intervention, with participants describing its ease of use, ability to track pain over time, and standalone utility. Engagement with the check-in feature appeared to support participants’ capacity for self-reflection on their pain experiences, a process that has been linked to greater self-awareness, autonomy, and a sense of ownership over pain self-management.^51^ This finding suggests that even brief, targeted digital tools may facilitate meaningful self-management, independent of more complex features. Moreover, given that improvements were observed in both groups, low-intensity interventions that create a sense of connection, engagement, or support may be sufficient to promote symptom improvement among AYA with chronic pain. This may be particularly relevant for youth already receiving high-intensity multidisciplinary care, where even minimal additional support could enhance self-management. Participants also emphasized the importance of age-appropriate and non-medical design. AYA valued that the app felt approachable and encouraging rather than overtly clinical, consistent with prior research indicating that visually engaging, youth-centered digital tools may promote sustained engagement.^52–54^

User feedback also highlighted several opportunities for program improvement and optimization (e.g., more mature aesthetic for older youth, addition of a medication tracker). While the check-in feature demonstrated high engagement, it differed from other intervention features (goals, library, and community forum) in keyways. It was brief, low effort to complete, and supported by daily push notification reminders, whereas other features required more time and active engagement without equivalent prompts. The integration of newer technologies could further enhance the personalization and adaptiveness of iCanCope for future users. For example, motivational interviewing–informed prompts or conversational agents (e.g., chatbots) could guide AYA toward positive behavior change by helping them identify relevant library content or further tailor the goal setting feature. These tools may empower AYA to translate their broader self-management goals into smaller, actionable steps aligned with their individual needs and readiness. The addition of new features, such as a gamified progress report to highlight symptom trends, areas for improvement, and goal attainment, may also reinforce engagement and motivation.

Although primary quantitative analyses did not demonstrate clinically significant effects, exploratory moderation analyses suggested that baseline insomnia may influence intervention effects over time. This finding raises the possibility that iCanCope may be more beneficial for AYA with sleep challenges. This possibility is consistent with prior work demonstrating larger intervention effects among AYA with co-occurring chronic pain and sleep challenges.^36–38^ Specifically, as these interventions, including the present study incorporate cognitive-behavioral strategies, they may support improvements in sleep, which in turn contribute to improvements in pain, given the well-established interplay between sleep and pain. Future research should consider evaluating iCanCope among AYA with more severe sleep issues.

### Strengths and limitations

The current study has several strengths. First, the user-centered design of the iCanCope program supported the development of a digital intervention that was highly acceptable to participants. Beyond a user-centered approach, AYA with chronic pain were meaningfully involved at multiple stages of the intervention lifecycle, including needs assessment, iterative design and refinement of app features, and usability testing.^12,13^ Consistent with best practices in digital health research, future intervention development should continue to prioritize the inclusion of end-users throughout the design and evaluation process.^52,55^ In alignment with recommendations for digital health trials, this study employed an active control condition, which strengthens internal validity and allows for a more rigorous assessment of intervention-specific effects; however, this also likely also reduced detectable between-group differences.^17–19^ Additionally, the use of an active control allowed participants to be blinded to the intervention allocation furthering study rigor. Finally, this study was pan-Canadian, recruiting participants from multiple pediatric and adult chronic pain clinics across Canada, increasing the representativeness of the sample and addressing limitations associated with single-center trials.

Several limitations should also be considered. First, the study employed a rolling design in which the trial began as a pilot randomized controlled trial to assess feasibility and preliminary signals of effectiveness and was subsequently extended to a full-scale randomized controlled trial once feasibility criteria were met. As a result, participants from both the pilot and extended phases were analyzed as part of a single, unified study, despite ongoing optimization of study procedures and workflows during the early phase. Importantly, no differences were observed in baseline pain or demographic characteristics between participants enrolled during the pilot and full-scale phases. However, extending directly to a full-scale trial without formally incorporating pilot findings into design refinements may have represented a missed opportunity to optimize the intervention and outcome selection. For example, although the inclusion of multiple clinical outcomes is recommended by the PedIMMPACT^56^ guidelines, the resulting questionnaire burden, combined with the requirement that measures be validated across a broad age range (15–25 years), may have limited sensitivity to change. Findings from the pilot phase could have informed a more focused selection of outcomes or a narrower age range for the full-scale trial. Second, baseline symptom severity was heterogeneous, resulting in a sample with moderate pain severity and preserved functioning in some domains. As such, recruitment did not align with periods of greatest clinical need for some AYA which may have limited the potential for additional benefit from the iCanCope program. Third, while the moderating effect of age was examined as a post-hoc analysis, the present study was likely underpowered, as the majority of participants were between 15–17 years of age, resulting in a relatively small young adult comparison group. Fourth, the present study consisted of predominately female-identifying and White AYA, as such, the findings may not generalize to AYA from marginalized or racialized communities. Future research should prioritize inclusive approaches to recruitment with increased efforts to engage more diverse populations, including racialized AYA, newcomers, AYA with lower digital access, and those historically underrepresented in pain research. Finally, part of the recruitment and data collection occurred during the COVID-19 pandemic, which may have influenced engagement with the intervention as increased digital demands, competing stressors, or pandemic-related disruptions may have attenuated the effects of the iCanCope program.

## Conclusion

The iCanCope with Pain program is one of the first digital interventions developed specifically for AYA with chronic pain to support pain self-management and related psychosocial symptoms. High levels of acceptability and engagement, together with positive qualitative findings speaking to improvements in agency and reflection, highlight the real-world value of symptom tracking and pain education for this population. Given the low cost and scalability of the intervention, the iCanCope program has been translated into French and will be publicly released. It has already been integrated as an evidence-based resource within the *Power over Pain* virtual stepped‑care platform for AYA with chronic pain.

## Supplementary Material

CONSORT_2010_Checklist_iCC CP.doc

Supplementary_Materials_April23_tracked.docx

Supplementary_Materials_April23_clean.docx

## Data Availability

The raw data will not be made available in order to protect against the possible identification of any patients who took part in the study. As per the institution's REB, any release of data requires contracts outlining the recipient of the data and purposed use of data.
