# Supplementary material for: Impacts of the iCanCope Digital Pain Self-Management Program Among Adolescents and Young Adults With Chronic Pain: A Randomized Controlled Trial
Source: Can J Pain. 2026 Oct 15;10(1):2717605. doi: 10.1080/24740527.2026.2717605 (PMC13679339; doi:10.1080/24740527.2026.2717605)
Supplement: Supplementary file 1 [file UCJP_A_2717605_SM6654.docx]

Appendix 1. Supplemental Methods

**iCanCope Intervention**

The iCanCope app includes four core components: pain intensity, activity limitations, mood, physical activity, sleep quality, energy. The symptom tracking and trending feature allows adolescents and young adults (AYA) to report their pain intensity, pain interference, mood, and sleep quality. This feature was designed to support AYA in recognizing and understanding patterns and potential triggers in their pain and daily functioning. Engagement with this feature was facilitated through daily push-notification reminders prompting AYA to complete check-ins, as well as the ability to view historical check-in data over time. The library provided curated, youth-friendly content including educational resources on chronic pain, skills-based strategies such as mindfulness and cognitive behavioural techniques, and practical guidance on self-advocacy and communication with healthcare providers. This content was designed to support in-the-moment learning and skill acquisition relevant to pain self-management. The goal-setting feature was designed to enhance self-efficacy, using principles of SMART goal setting, AYA were supported in creating structured, personalized goals related to improving pain management and daily functioning. Periodic reminders to set goals, along with positive feedback on progress toward goal completion, were incorporated to encourage continued engagement. The community forum provided AYA with a structured and moderated space to interact with other young people living with chronic pain. To foster engagement and discussion, the study team posted a new discussion prompt each week (e.g., “What’s your favorite distraction from pain?”). AYA were able to respond to prompts and react to other users’ posts using a “favourite” function, facilitating peer connection and shared experiences.

**Missing Data**

Missing outcome data was assessed across the three study time points to identify patterns of missingness and to inform assumptions regarding the missing data mechanism. Participants were categorized based on completeness of outcome data as follows: (1) participants with complete outcome data at all three time points (T1 to T3); (2) participants missing outcome data at T3 only (excluding those in the pilot studies) or T2; and (3) participants missing outcome data at both T2 and T3. To examine whether outcome missingness was associated with baseline factors, demographic and clinical characteristics were compared across these groups. Clinically meaningful differences were not found (Supplementary Table 1). These findings support the assumption of missing at random, participants with incomplete follow-up data were retained in all analyses.

**Sensitivity Analysis**

Two sensitivity analyses were completed, the first to explore whether there were differences in demographic and disease characteristics between participants initially recruited in the pilot trial and those who completed the full-scale trial. Differences were explored visually; however formal statistical testing was not performed. Secondly, a per-protocol analysis was completed whereby participants were reassigned to allocation group based on their engagement with the app (i.e., those who did not engage with the app were moved to the control group) to assess the effect of receiving the intervention as intended. Subsequently, the aforementioned analysis was re-run, using the same alpha levels as the primary analysis. Which was a significance level of 0.05 for the primary outcome and a Bonferroni-adjusted alpha level of was used to maintain an overall level of 0.0031 for all secondary outcomes.

**Post-hoc analysis plan**

Two exploratory post-hoc analysis was performed, the first to understand the impact of treatment expectancy (TE, measured only at baseline) and the second to understand whether the age and insomnia moderated the effect of the intervention on pain intensity and pain interference. First to assess whether TE improved the ability to detect treatment effects, the same linear mixed models were re-ran, using the same significance levels as the primary analysis with the addition of TE and interaction term of TE and 3-level time indicator (T2, T3 vs T1). Change in the estimate of intervention variable would support the conclusion that TE improved the ability to detect treatment efficacy. Secondly, to understand the main effect of TE over time regardless of allocation group, the interaction term of TE and the 3-level indicator for assessment time points were reported using the same model described above. A positive effect estimate suggested that higher score in the respective outcome corresponded to higher TE.

For the moderation analysis, age was dichotomized as adolescents (15–17-year-olds) and young adults (18+), and insomnia was dichotomized as clinical insomnia (ISI ≥ 15) and no clinical insomnia as (ISI < 15). Moderation was assessed by including a 3-way interaction of age or insomnia with the 3-level time indicator and group in the linear mixed model, with pain intensity or pain interference as the outcome respectively. A significant three-way interaction (alpha at 0.05) was interpreted as evidence for moderation.

Supplementary Table 1. Adolescent and young adult demographics and pain characteristics by missingness category.

|  | **Missing only T3^a^ or T2 (n=41)** | **Missing T2 and T3 (n=31)** | **Complete data (n=153)** |
| --- | --- | --- | --- |
| Age in years, median (IQR) | 16 (15 – 18) | 17 (16 – 17) | 17 (15 – 16) |
| Gender, n(%) |  |  |  |
| *Female* | 34 (85%) | 23 (79%) | 134 (90%) |
| *Male* | 6 (15%) | 6 (21%) | 13 (9%) |
| *Non-binary* | 0 | 0 | 2 (1%) |
| *Missing* | 1 | 4 | 4 |
| Race, n(%) |  |  |  |
| *Indigenous* | 1 (2%) | 0 | 3 (2%) |
| *Arab/West Asian* | 1 (2%) | 0 | 1 (1%) |
| *Asian (Chinese, Filipino, Korean, South Asian, South East Asian)* | 0 | 1 (4%) | 9 (7%) |
| *Black* | 0 | 1 (4%) | 2 (1%) |
| *Latin American* | 0 | 1 (4%) | 1 (1%) |
| *White* | 34 (85%) | 23 (79%) | 118 (78%) |
| *Mixed Race* | 2 (5%) | 3 (10%) | 15 (10%) |
| *Other* | 2 (5%) | 0 | 2 (1%) |
| *Missing* | 1 | 2 | 2 |
| Location of Pain^b^ |  |  |  |
| *Abdominal* | 9 (22%) | 7 (27%) | 52 (33%) |
| *Facial* | 5 (12%) | 1 (4%) | 15 (9%) |
| *Headache* | 17 (41%) | 7 (27%) | 66 (42%) |
| *Low back* | 15 (37%) | 9 (35%) | 72 (45%) |
| *Musculoskeletal* | 18 (44%) | 10 (38%) | 66 (42%) |
| *Neuropathic* | 9 (22%) | 4 (15%) | 42 (26%) |
| *Pelvic* | 9 (22%) | 4 (15%) | 17 (11%) |
| *Other* | 8 (20%) | 11 (42%) | 47 (30%) |
| Number of Pain Sites, median (IQR) | 2 (1 – 3) | 2 (1 – 3) | 2 (1 – 4) |
| Duration of pain in years, median (IQR) | 4.5 (2 – 6.75) | 4 (2 – 7) | 4 (2 – 7) |

^a^ Pilot participants are not included in this category. ^b^ Participants were able to select multiple pain areas.

Supplementary Table 2. Baseline characteristics of participants enrolled in the pilot study and full-scale randomized controlled trial.

|  | **Pilot (n=72)** | **Full-scale RCT (n=255)** |
| --- | --- | --- |
| Age in years, median (IQR) | 16 (15 – 17) | 17 (16 – 18) |
| Gender, n(%) |  |  |
| *Female* | 61 (86%) | 191 (88%) |
| *Male* | 10 (14%) | 25 (11%) |
| *Non-binary* | 0 (0%) | 2 (1%) |
| *Missing* | 1 | 7 |
| Race, n(%) |  |  |
| *Indigenous* | 1 (2%) | 4 (2%) |
| *Arab/West Asian* | 2 (3%) | 2 (1 %) |
| *Asian (Chinese, Filipino, Korean, South Asian, South East Asian)* | 6 (8%) | 10 (4%) |
| *Black* | 1 (2%) | 3  (2%) |
| *Latin American* | 0  (0%) | 2  (1%) |
| *White* | 44 (70%) | 175 (78%) |
| *Mixed Race* | 6  (10%) | 20 (9%) |
| *Other* | 3 (5%) | 4  (2%) |
| *Missing* | 9 | 5 |
| Location of Pain^a^ |  |  |
| *Abdominal* | 20 (28%) | 68 (30%) |
| *Facial* | 6 (8%) | 21 (9%) |
| *Headache* | 33 (46%) | 89 (40%) |
| *Low back* | 27 (38%) | 95 (42%) |
| *Musculoskeletal* | 22 (31%) | 94 (42%) |
| *Neuropathic* | 15 (21%) | 55 (24%) |
| *Pelvic* | 12 (17%) | 30 (13%) |
| *Other* | 22 (31%) | 66 (29%) |
| Number of Pain Sites, median (IQR) | 2 (1 – 3) | 2 (1 – 3) |
| Duration of pain in years, median (IQR) | 4 (2.46 - 6.50) | 4 (2 – 7) |

^a^ Participants were able to select multiple pain areas.

Supplementary Table 3. Mean adolescent and young adult reported primary and secondary outcomes across study time points.

|  | **Intervention (n=147)** | **Control (n=150)** |
| --- | --- | --- |
| Pain Interference (range 0 – 10, higher scores indicate greater pain interference) | | |
| T1 – Baseline | 5.4 (2.2) | 5.7 (1.9) |
| T2 – 2 months | 4.8 (2.1) | 5.2 (2.0) |
| T3 – 6 months | 4.7 (2.3) | 5.1 (2.3) |
| Average Pain Intensity (range 0 – 10, higher scores indicate greater pain intensity) | | |
| T1 – Baseline | 5.4 (1.8) | 5.5 (1.6) |
| T2 – 2 months | 5.0 (1.6) | 5.3 (1.8) |
| T3 – 6 months | 4.7 (1.9) | 5.3 (1.9) |
| Pain Self-Efficacy (range 0 – 60, higher scores suggesting greater levels of confidence in dealing with pain) | | |
| T1 – Baseline | 33.6 (12.8) | 31.1 (11.8) |
| T2 – 2 months | 36.3 (12.4) | 32.1 (11.7) |
| T3 – 6 months | 35.7 (14.7) | 32.7 (13.7) |
| Social Support (range 0 – 20, higher scores suggesting greater social support from friends) | | |
| T1 – Baseline | 12.5 (5.3) | 12.8 (4.9) |
| T2 – 2 months | 12.6 (5.6) | 12.6 (5.3) |
| T3 – 6 months | 12.6 (5.9) | 13.3 (5.3) |
| Anxiety (range 0 – 21, higher scores suggest more anxiety symptoms, scores >11 represent clinical anxiety) | | |
| T1 – Baseline | 9.9 (3.1) | 10.1 (2.7) |
| T2 – 2 months | 9.7 (3.3) | 10.0 (2.8) |
| T3 – 6 months | 9.7 (2.8) | 10.3 (2.9) |
| Depression (range 0 – 21, higher scores suggest more depressive symptoms, scores >11 represent clinical depression) | | |
| T1 – Baseline | 5.9 (2.5) | 6.0 (2.4) |
| T2 – 2 months | 5.9 (2.5) | 6.2 (2.6) |
| T3 – 6 months | 5.6 (2.9) | 6.2 (2.5) |
| Insomnia (range 0 – 28, higher scores suggest worse sleep outcomes, scores ≥ 15 represent clinical insomnia) | | |
| T1 – Baseline | 13.5 (6.6) | 12.2 (6.4) |
| T2 – 2 months | 12.9 (6.5) | 13.1 (6.5) |
| T3 – 6 months | 12.0 (6.7) | 12.1 (7.1) |
| SF-36 Physical Functioning Domain (range 0 – 100, higher scores better) | | |
| T1 – Baseline | 55.0 (24.2) | 51.6 (25.9) |
| T2 – 2 months | 59.0 (24.8) | 55.8 (26.3) |
| T3 – 6 months | 55.8 (26.5) | 55.7 (24.2) |
| SF-36 Physical Limitations Domain (range 0 – 100, higher scores better) | | |
| T1 – Baseline | 29.8 (35.9) | 25.8 (32.9) |
| T2 – 2 months | 40.0 (40.7) | 29.6 (34.9) |
| T3 – 6 months | 38.5 (39.6) | 29.1 (36.3) |
| SF-36 Limitations of Emotional Health Domain (range 0 – 100, higher scores better) | | |
| T1 – Baseline | 44.7 (39.2) | 44.4 (39.9) |
| T2 – 2 months | 46.6 (41.7) | 42.6 (40.7) |
| T3 – 6 months | 52.1 (41.5) | 43.4 (42.5) |
| SF-36 Energy and Fatigue Domain (range 0 – 100, higher scores better) | | |
| T1 – Baseline | 31.7 (20.2) | 30.5 (20.4) |
| T2 – 2 months | 34.6 (22.4) | 32.3 (20.5) |
| T3 – 6 months | 35.8 (23.8) | 31.6 (20.9) |
| SF-36 Emotional Wellbeing Domain (range 0 – 100, higher scores better) | | |
| T1 – Baseline | 55.4 (18.3) | 54.4 (18.5) |
| T2 – 2 months | 53.2 (19.8) | 54.0 (15.7) |
| T3 – 6 months | 55.7 (19.7) | 54.9 (19.2) |
| SF-36 Social Functioning Domain (range 0 – 100, higher scores better) | | |
| T1 – Baseline | 53.7 (25.7) | 51.0 (26.1) |
| T2 – 2 months | 56.9 (27.0) | 54.9 (25.7) |
| T3 – 6 months | 57.7 (28.1) | 53.8 (25.9) |
| SF-36 Pain Domain (range 0 – 100, higher scores better) | | |
| T1 – Baseline | 38.1 (18.1) | 34.3 (18.0) |
| T2 – 2 months | 42.6 (19.8) | 39.6 (18.3) |
| T3 – 6 months | 43.9 (21.2) | 40.5 (19.4) |
| SF-36 General Health Domain (range 0 – 100, higher scores better) | | |
| T1 – Baseline | 33.0 (17.3) | 33.1 (17.2) |
| T2 – 2 months | 35.6 (18.1) | 33.2 (17.1) |
| T3 – 6 months | 35.7 (19.2) | 34.3 (17.2) |
| Pain Knowledge - About Pain Domain (range 1 – 10, higher scores suggest increase in knowledge) | | |
| T1 – Baseline | 5.1 (2.2) | 5.3 (2.3) |
| T2 – 2 months | 5.8 (2.2) | 5.7 (2.1) |
| T3 – 6 months | 6.6 (1.9) | 6.3 (2.2) |
| Pain Knowledge – Managing Pain Domain (range 1 – 10, higher scores suggest increase in knowledge) | | |
| T1 – Baseline | 4.9 (1.8) | 5.1 (1.9) |
| T2 – 2 months | 5.5 (1.8) | 5.6 (1.8) |
| T3 – 6 months | 6.1 (1.8) | 6.2 (1.9) |
| Patient Global Impression of Change (range 1 – 7, higher scores suggest greater perceived change) | | |
| T1 – Baseline | 2.6 (1.6) | 2.5 (1.6) |
| T2 – 2 months | 2.9 (1.8) | 2.7 (1.6) |
| T3 – 6 months | 3.3 (1.9) | 3.0 (1.8) |

Supplementary Table 4. Adolescent and young adults reported primary and secondary outcomes, per-protocol linear mixed models.

|  |  |  |  |  |  |  |  | SF-36 Domains | | | | | | | | MEPs Domains | |
| --- | --- | --- | --- | --- | --- | --- | --- | --- | --- | --- | --- | --- | --- | --- | --- | --- | --- |
|  | Pain Interference | Average Pain | Pain Self-efficacy | Social Support | Anxiety | Depression | Insomnia | Physical Functioning | Physical Limitations | Limitations of Emotional Health | Energy and Fatigue | Emotional Wellbeing | Social Functioning | Pain | General Health | About Pain | Managing Pain |
| **Intervention** | -0.40 | -0.15 | 3.07 | 0.08 | -0.16 | -0.14 | 0.43 | 3.23 | 3.88 | -0.81 | 1.24 | 0.54 | 2.00 | 3.77 | 0.03 | -0.27 | -0.17 |
| *p-value* | 0.11 | 0.48 | 0.04 | 0.90 | 0.64 | 0.64 | 0.58 | 0.28 | 0.37 | 0.86 | 0.62 | 0.89 | 0.52 | 0.09 | 0.99 | 0.32 | 0.43 |
| *Lower CI* | -0.89 | -+0.55 | 0.15 | -1.30 | -0.84 | -0.74 | -1.09 | -2.63 | -4.56 | -10.18 | -3.65 | -3.70 | -4.08 | -0.62 | -4.02 | -0.77 | -0.60 |
| *Upper CI* | 0.10 | 0.26 | 6.00 | 1.14 | 0.52 | 0.45 | 1.95 | 9.09 | 12.3 | 8.55 | 6.13 | 4.79 | 8.07 | 8.16 | 4.09 | 0.24 | 0.26 |
| **Time 2** | **-0.58** | -0.4 | 1.18 | -0.24 | -0.05 | 0.18 | -0.19 | 4.21 | 3.44 | -2.00 | 1.72 | -0.39 | 3.55 | **5.19** | 0.14 | 0.39 | **0.54** |
| *p-value* | **p < 0.05** | 0.31 | 0.22 | 0.53 | 0.85 | 0.42 | 0.68 | 0.09 | 0.31 | 0.56 | 0.23 | 0.78 | 0.10 | **p < 0.003** | 0.89 | 0.01 | **p < 0.003** |
| *Lower CI* | **-0.90** | -0.41 | -0.71 | -0.97 | -0.58 | -0.26 | -1.13 | 1.05 | -3.14 | -8.66 | -1.09 | -3.12 | -0.63 | **1.85** | -1.86 | 0.09 | **0.28** |
| *Upper CI* | **-0.25** | 0.13 | 3.06 | 0.49 | 0.48 | 0.62 | 0.74 | 7.36 | 10.0 | 4.67 | 4.52 | 2.33 | 7.73 | **8.52** | 2.14 | 0.68 | **0.81** |
| **Time 3** | **-0.64** | -0.20 | 2.03 | 0.22 | 0.13 | 0.27 | -0.77 | 4.65 | 3.22 | -0.69 | 1.22 | 0.40 | 3.39 | **6.55** | 1.85 | **0.98** | **1.05** |
| *p-value* | **p < 0.05** | 0.23 | 0.09 | 0.63 | 0.68 | 0.32 | 0.19 | 0.02 | 0.43 | 0.87 | 0.49 | 0.81 | 0.20 | **p < 0.003** | 0.14 | **p < 0.003** | **p < 0.003** |
| *Lower CI* | **-1.04** | -0.53 | -0.30 | -0.68 | -0.51 | -0.26 | -1.92 | 0.76 | -4.80 | -8.84 | -2.22 | -2.94 | -1.74 | **2.49** | -0.62 | **0.62** | **0.72** |
| *Upper CI* | **-0.24** | 0.13 | 4.52 | 1.12 | 0.77 | 0.81 | 0.39 | 8.54 | 11.25 | 7.46 | 4.67 | 3.75 | 8.51 | **10.60** | 4.32 | **1.35** | **1.37** |
| **Intervention x Time 2** | 0.09 | -0.15 | 0.74 | 0.10 | -0.12 | -0.04 | -0.55 | -0.46 | 6.58 | 3.90 | 0.88 | -1.81 | -0.16 | -0.61 | 2.25 | 0.36 | 0.01 |
| *p-value* | 0.9 | 0.46 | 0.59 | 0.85 | 0.75 | 0.90 | 0.43 | 0.84 | 0.18 | 0.43 | 0.67 | 0.37 | 0.96 | 0.80 | 0.13 | 0.10 | 0.85 |
| *Lower CI* | -0.37 | -0.53 | -1.98 | -1.96 | -0.88 | -0.68 | -1.90 | -5.00 | -2.94 | -5.74 | -3.16 | -5.75 | -6.21 | -5.42 | -0.63 | -0.07 | -0.37 |
| *Upper CI* | 0.56 | 0.24 | 3.46 | 1.16 | 0.63 | 0.59 | 0.80 | 4.09 | 16.10 | 13.53 | 4.92 | 2.13 | 5.88 | 4.20 | 5.14 | 0.79 | 0.39 |
| **Intervention x Time 3** | 0.04 | -0.52 | -1.04 | -0.63 | -0.46 | -0.41 | -1.91 | -1.59 | 5.13 | 7.39 | 2.72 | -1.72 | 0.85 | 0.35 | 0.84 | 0.31 | 0.04 |
| *p-value* | 0.90 | 0.03 | 0.54 | 0.33 | 0.32 | 0.29 | 0.27 | 0.57 | 0.38 | 0.21 | 0.28 | 0.48 | 0.82 | 0.90 | 0.64 | 0.25 | 0.88 |
| *Lower CI* | -0.53 | -0.99 | -4.32 | -1.92 | -1.37 | -1.17 | -2.56 | -7.15 | -6.32 | -4.25 | -2.19 | -6.50 | -6.47 | -5.44 | -2.68 | -0.21 | -0.43 |
| *Upper CI* | 0.60 | -0.05 | 2.26 | 0.65 | 0.45 | 0.35 | 0.73 | 3.96 | 16.60 | 19.03 | 7.56 | 3.06 | 8.17 | 6.14 | 4.38 | 0.83 | 0.50 |

Note: Bolded values indicate statistical significance at an alpha level of 0.05 for the primary outcome (pain interference) and a Bonferroni-adjusted alpha of 0.0031 for secondary outcomes.

Supplementary Table 5. Effect of youth pretreatment treatment expectations on primary and secondary outcomes.

|  |  |  |  |  |  |  |  | SF-36 Domains | | | | | | | | MEPs Domains | |
| --- | --- | --- | --- | --- | --- | --- | --- | --- | --- | --- | --- | --- | --- | --- | --- | --- | --- |
|  | Pain Interference | Average Pain | Pain Self-efficacy | Social Support | Anxiety | Depression | Insomnia | Physical Functioning | Physical Limitations | Limitations of Emotional Health | Energy and Fatigue | Emotional Wellbeing | Social Functioning | Pain | General Health | About Pain | Managing Pain |
| **Intervention** | -0.49 | -0.19 | 2.85 | -0.36 | -0.06 | -0.01 | 0.07 | 3.56 | 4.18 | 0.75 | 1.30 | 0.04 | 2.82 | 4.61 | -1.12 | -0.25 | -0.16 |
| *p-value* | 0.05 | 0.35 | 0.06 | 0.56 | 0.85 | 0.99 | 0.93 | 0.24 | 0.33 | 0.88 | 0.60 | 0.98 | 0.37 | 0.04 | 0.59 | 0.34 | 0.48 |
| *Lower CI* | -0.98 | -0.60 | -0.08 | -1.59 | -0.75 | -0.59 | -1.45 | -2.35 | -4.25 | -8.68 | -3.60 | -4.21 | -3.29 | 0.21 | -5.17 | -0.75 | -0.59 |
| *Upper CI* | 0.01 | 0.21 | 5.78 | 0.86 | 0.62 | 0.58 | 1.58 | 9.48 | 12.62 | 10.18 | 6.20 | 4.30 | 8.94 | 9.02 | 2.94 | 0.26 | 0.28 |
| **Time 2** | -0.23 | 0.21 | -1.99 | -1.60 | -0.87 | 0.75 | 0.65 | 0.19 | -5.75 | -13.73 | 3.16 | 4.44 | -0.71 | -2.61 | -0.65 | -0.08 | 0.40 |
| *p-value* | 0.55 | 0.51 | 0.38 | 0.07 | 0.17 | 0.15 | 0.56 | 0.96 | 0.46 | 0.09 | 0.33 | 0.17 | 0.89 | 0.51 | 0.78 | 0.82 | 0.21 |
| *Lower CI* | -0.99 | -0.42 | -6.43 | -3.32 | -2.12 | -0.28 | -1.56 | -7.20 | -21.12 | -29.36 | -3.27 | -1.90 | -10.47 | -10.32 | -5.29 | -0.77 | -0.22 |
| *Upper CI* | 0.53 | 0.85 | 2.45 | 0.13 | 0.38 | 1.78 | 2.85 | 7.59 | 9.63 | 1.90 | 9.60 | 10.78 | 9.05 | 5.10 | 3.99 | 0.61 | 1.01 |
| **Time 3** | 0.08 | -0.22 | -3.97 | -0.91 | -0.30 | 0.74 | 0.36 | 1.05 | -6.23 | 6.03 | -8.30 | 7.30 | 0.34 | -6.12 | 4.04 | 0.22 | 0.47 |
| *p-value* | 0.87 | 0.57 | 0.15 | 0.40 | 0.70 | 0.24 | 0.80 | 0.82 | 0.52 | 0.54 | 0.04 | 0.07 | 0.96 | 0.20 | 0.17 | 0.62 | 0.22 |
| *Lower CI* | -0.85 | -1.00 | -9.42 | -3.02 | -1.82 | -0.51 | -2.35 | -8.11 | -25.00 | -13.14 | -16.24 | -0.50 | -11.65 | -15.54 | -1.72 | -0.63 | -0.28 |
| *Upper CI* | 1.01 | 0.55 | 1.47 | 1.21 | 1.22 | 2.00 | 3.07 | 10.22 | 12.54 | 25.19 | -0.36 | 15.11 | 12.32 | 3.30 | 9.80 | 1.07 | 1.23 |
| **Intervention x Time 2** | 0.12 | -0.11 | 0.53 | -0.01 | -0.20 | -0.03 | -0.41 | -0.91 | 5.35 | 1.74 | 1.21 | -1.22 | -1.12 | -1.63 | 2.20 | 0.29 | -0.03 |
| *p-value* | 0.62 | 0.57 | 0.70 | 0.98 | 0.61 | 0.91 | 0.56 | 0.70 | 0.27 | 0.73 | 0.55 | 0.54 | 0.72 | 0.51 | 0.13 | 0.20 | 0.89 |
| *Lower CI* | -0.35 | -0.51 | -2.21 | -1.07 | -0.97 | -0.67 | -1.77 | -5.51 | -4.22 | -7.99 | -2.80 | -5.18 | -7.19 | -6.43 | -0.68 | -0.15 | -0.41 |
| *Upper CI* | 0.59 | 0.28 | 3.27 | 1.05 | 0.57 | 0.60 | 0.95 | 3.68 | 14.93 | 11.47 | 5.21 | 2.73 | 4.96 | 3.17 | 5.08 | 0.72 | 0.35 |
| **Intervention x Time 3** | 0.04 | -0.52 | -1.47 | -0.55 | -0.49 | -0.54 | -0.76 | -2.20 | 5.28 | 6.95 | 2.07 | -0.46 | 0.34 | -1.14 | 1.10 | 0.23 | -0.02 |
| *p-value* | 0.90 | 0.03 | 0.39 | 0.41 | 0.31 | 0.17 | 0.37 | 0.45 | 0.37 | 0.25 | 0.41 | 0.85 | 0.93 | 0.70 | 0.55 | 0.40 | 0.92 |
| *Lower CI* | -0.54 | -0.99 | -4.82 | -1.86 | -1.42 | -1.31 | -2.43 | -7.87 | -6.35 | -4.92 | -2.85 | -5.29 | -7.08 | -6.98 | -2.46 | -0.30 | -0.49 |
| *Upper CI* | 0.61 | -0.04 | 1.89 | 0.75 | 0.45 | 0.24 | 0.91 | 3.48 | 16.91 | 18.81 | 6.98 | 4.37 | 7.77 | 4.69 | 4.66 | 0.76 | 0.45 |
| **Treatment Expectancy** | 0.10 | 0.11 | -0.04 | 0.21 | -0.19 | -0.13 | 0.41 | -0.10 | -0.23 | -0.51 | 0.12 | 1.22 | -0.02 | -0.85 | 1.32 | -0.03 | -0.03 |
| *p-value* | 0.10 | 0.03 | 0.90 | 0.18 | 0.03 | 0.08 | 0.03 | 0.89 | 0.83 | 0.66 | 0.85 | 0.02 | 0.98 | 0.12 | 0.01 | 0.62 | 0.53 |
| *Lower CI* | -0.02 | 0.01 | -0.76 | -0.09 | -0.35 | -0.27 | 0.04 | -1.54 | -2.29 | -2.82 | -1.08 | 0.18 | -1.51 | -1.92 | 0.33 | -0.15 | -0.14 |
| *Upper CI* | 0.22 | 0.21 | 0.67 | 0.51 | -0.02 | 0.02 | 0.78 | 1.34 | 1.83 | 1.79 | 1.31 | 2.25 | 1.47 | 0.23 | 2.30 | 0.09 | 0.07 |
| **Treatment Expectancy x Time 2** | -0.06 | -0.06 | 0.53 | 0.23 | 0.14 | -0.09 | -0.14 | 0.67 | 1.54 | 1.97 | -0.24 | -0.81 | 0.71 | 1.31 | 0.13 | 0.08 | 0.03 |
| *p-value* | 0.31 | 0.22 | 0.12 | 0.09 | 0.15 | 0.25 | 0.40 | 0.24 | 0.20 | 0.10 | 0.63 | 0.10 | 0.35 | 0.03 | 0.72 | 0.14 | 0.60 |
| *Lower CI* | -0.17 | -0.16 | -0.14 | -0.03 | -0.05 | -0.25 | -0.48 | -0.45 | -0.79 | -0.41 | -1.22 | -1.78 | -0.77 | 0.14 | -0.57 | -0.03 | -0.07 |
| *Upper CI* | 0.06 | 0.04 | 1.20 | 0.49 | 0.33 | 0.06 | 0.19 | 1.79 | 3.88 | 4.34 | 0.73 | 0.15 | 2.20 | 2.48 | 0.83 | 0.18 | 0.12 |
| **Treatment Expectancy x Time 2** | -0.11 | 0.00 | 0.97 | 0.18 | 0.08 | -0.06 | -0.16 | 0.59 | 1.43 | -1.10 | 1.45 | -1.22 | 0.41 | 2.02 | -0.41 | 0.13 | 0.10 |
| *p-value* | 0.12 | 0.99 | 0.02 | 0.27 | 0.49 | 0.54 | 0.43 | 0.40 | 0.32 | 0.45 | 0.02 | 0.04 | 0.65 | 0.01 | 0.35 | 0.05 | 0.09 |
| *Lower CI* | -0.25 | -0.12 | 0.16 | -0.14 | -0.15 | -0.25 | -0.57 | -0.78 | -1.38 | -3.97 | 0.27 | -2.39 | -1.38 | 0.61 | -1.27 | 0.00 | -0.02 |
| *Upper CI* | 0.03 | 0.11 | 1.78 | 0.49 | 0.31 | 0.13 | 0.24 | 1.96 | 4.24 | 1.77 | 2.64 | -0.06 | 2.20 | 3.43 | 0.45 | 0.26 | 0.21 |

Note: Bolded values indicate statistical significance at an alpha level of 0.05 for the primary outcome (pain interference) and a Bonferroni-adjusted alpha of 0.0031 for secondary outcomes.

Supplementary Table 6. Moderating effect of age and insomnia on pain intensity and pain interference.

|  | Study Time Point | Age | | Insomnia | |
| --- | --- | --- | --- | --- | --- |
|  |  | β (95% CI) | p-value | β (95% CI) | p-value |
| Pain intensity | T2 – 2 months | 0.17  (-0.70, 1.0) | 0.695 | 0.22  (0.56, 0.99) | 0.581 |
|  | T3 – 6 months | 0.77  (-0.27, 1.82) | 0.148 | -1.01  (-1.96, -0.06) | 0.038 |
| Pain interference | T2 – 2 months | -0.03  (-1.10, 1.04) | 0.957 | -0.61  (-1.53, 0.32) | 0.201 |
|  | T3 – 6 months | 0.18  (-1.09, 1.44) | 0.783 | -2.06  (-3.19, -0.93) | 0.000 |

Note. The beta coefficient represents the three-way interaction of Intervention Group × Time × Age Group/Insomnia Status. It reflects whether there is a difference in change over time between the intervention and control groups by age group or insomnia status. A significant coefficient means that the intervention effects over time differ between AYA with and without insomnia.

Supplementary Figure 1. Distribution of Check-in Compliance Categories by Study Group.
